# Application of biomaterials in vascularization of cardiac organoids

**DOI:** 10.1016/j.isci.2025.113531

**Published:** 2025-09-08

**Authors:** Jun Liu, Yaxing Feng, Peng Qu, Yunbo Luo, Jiao Shi, Cui Ma, Qi Liang, Long Zhao, Gang Li, Bo Yang, Panke Cheng

**Affiliations:** 1 Department of Radiotherapy, The Affiliated Hospital of Southwest Medical University, Luzhou 646000, China; 2 Institute of Cardiovascular Diseases & Department of Cardiology, Sichuan Provincial People’s Hospital, School of Medicine, University of Electronic Science and Technology of China, Chengdu 610072, China; 3 Department of Breast Surgery, Plastic Surgery, Sichuan Cancer Hospital & Institute, Sichuan Cancer Center, School of Medicine, University of Electronic Science and Technology of China, Chengdu, Sichuan Province, China; 4 Department of Neurosurgery, Affiliated Hospital of North Sichuan Medical College, Nanchong, Sichuan 637000, China; 5 Core Laboratory, School of Medicine, Sichuan Provincial People’s Hospital Affiliated to University of Electronic Science and Technology of China, Chengdu 610072, China; 6 Department of Clinical Laboratory, Affiliated Hospital of North Sichuan Medical College, School of Laboratory Medicine, Translational Medicine Research Center, North Sichuan Medical College, Nanchong 637007, Sichuan, China; 7 Department of Mathematics, Army Medical University, Chongqing 400038, China; 8 Ultrasound Medicine and Computational Cardiology Key Laboratory of Sichuan Province, Chengdu 610072, China

**Keywords:** Biological sciences, Materials science

## Abstract

Cardiac organoids (COs) are platforms for disease modeling and regenerative medicine, yet inadequate vascularization still limits their function and longevity. This review examines how biomaterial properties—porosity, elasticity, surface chemistry, and bioactivity—regulate endothelial cell behavior and vascular network formation, thereby shaping progress in organoid vascularization. We survey natural, synthetic, and composite systems (e.g., collagen, fibrin, polyethylene glycol [PEG] hydrogels, and poly(ε-caprolactone) [PCL] scaffolds) used to promote vascularization. We also summarize fabrication strategies to improve perfusion, including micro/nanopatterning, scaffold design, and delivery of pro-angiogenic factors, and we outline technical routes for spatially patterned vasculature such as three-dimensional (3D) bioprinting and microfluidics. Ongoing challenges include degradation mismatch and incomplete integration. Future work should emphasize stimuli-responsive and bioactive materials, as well as standardized, scalable scaffold platforms, to strengthen reproducibility and translational efficiency. These directions will help optimize the vascular microenvironment of organoids, enhance functional maturation, and broaden applications in disease modeling, drug evaluation, and regenerative repair.

## Graphical abstract

**Figure:**
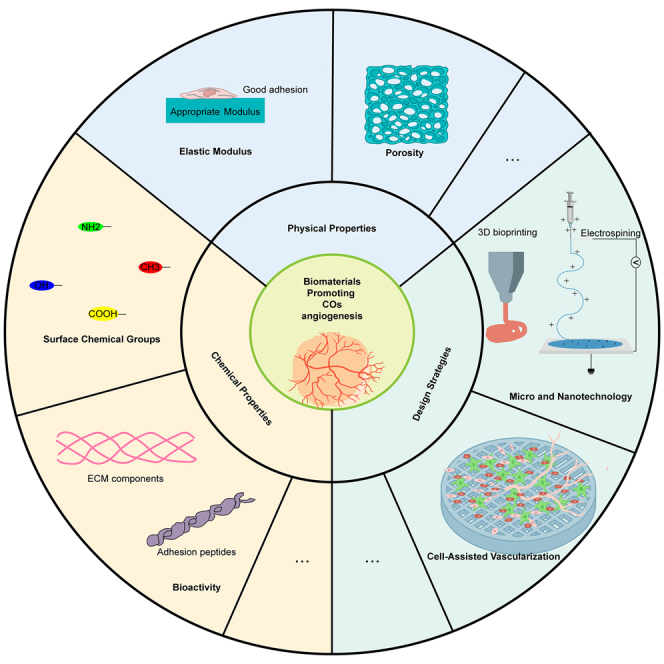

## Introduction

Cardiac organoids (COs) are miniature heart-like tissues derived from pluripotent stem cells (PSCs), capable of recapitulating key aspects of cardiac anatomy, cellular composition, and functional characteristics to a certain extent. They have been widely utilized in cardiac disease modeling, drug screening, and regenerative medicine research.^1^ By using human PSCs, COs with central cavity structures can be generated to simulate disease progression, explore underlying mechanisms, and evaluate drug-induced cardiotoxicity and regenerative potential.^2^ For instance, organoids can be employed to assess the effects of drugs on cardiac contractile function, thereby informing drug safety evaluations.^3^^,^^4^ In the field of cardiac regenerative medicine, COs also hold great promise. Patient-specific COs can be established using induced pluripotent stem cell (iPSC) technology, advancing the development of personalized medicine. These organoids offer unique opportunities to investigate patient-specific disease mechanisms and assess tailored therapeutic strategies.^5^ Furthermore, COs provide a powerful platform to study molecular and cellular changes during cardiac development, facilitating elucidation of the mechanisms underlying heart formation and congenital heart diseases.^6^ They are also applicable in developmental toxicity assessments—for example, studies have shown that low-dose cadmium exposure can impair mesoderm formation, thereby affecting cardiomyocyte differentiation and cardiac development, highlighting their utility in toxicological research.^7^ COs have been extensively applied in cardiovascular disease (CVD) research, including analyses of gene expression, cardiomyocyte size, and contractile function, thereby aiding in the elucidation of pathological mechanisms and evaluation of potential therapeutic strategies.^8^ Additionally, organoids can be leveraged to identify regulatory single-nucleotide polymorphisms (SNPs) associated with CVD and explore their correlation with gene expression.

However, insufficient vascularization remains a critical bottleneck limiting the application of COs, as it hampers nutrient delivery, metabolic waste removal, and functional maintenance.^9^ Studies have demonstrated that organoids lacking perfusable vascular systems often develop hypoxic cores, resulting in central necrosis and functional impairment—major constraints on their size expansion and long-term culture viability.^10^^,^^11^ Therefore, vascularization plays a central role in maintaining physiological function and supporting prolonged culture of COs. A well-established vascular network provides essential oxygen and nutrients; eliminates metabolic by-products; and facilitates organoid growth, functional maturation, and stability.^12^ Engineering a microvascular system that resembles native cardiac tissue not only helps recreate a physiologically relevant microenvironment but also enhances cardiomyocyte maturation and contractile function, thereby expanding the potential applications of COs in disease modeling, drug discovery, and regenerative medicine.^13^ Consequently, developing exogenous strategies to remodel the vascular microenvironment within COs has emerged as a key research focus.

Biomaterials are considered one of the most promising tools to achieve this goal due to their unique advantages in cellular support, microenvironmental regulation, and promotion of angiogenesis. The physical and chemical properties of biomaterials can modulate cellular behaviors such as adhesion, proliferation, and differentiation. Certain biomaterials, such as silk fibroin (SF), have gained significant attention owing to their excellent biocompatibility and tunable mechanical properties.^14^ Some biomaterials also possess inherent pro-angiogenic potential.^15^ Moreover, the combination of biomaterials with angiogenic factors or cell-based therapies can further enhance their capacity to induce vascularization.^16^ For example, the incorporation of endothelial cells or vascular endothelial growth factor (VEGF) into biomaterials can promote endothelial cell migration and tubular structure formation, accelerating the establishment of vascular networks.^17^

Biomaterials can also mimic the *in vivo* microenvironment to support the maturation of COs. By incorporating extracellular matrix (ECM) components such as collagen or laminin, biomaterials can provide cardiac cells with a more physiologically relevant growth environment.^18^ In addition, the application of advanced fabrication technologies such as three-dimensional (3D) printing, electrospinning, and microfluidics allows for precise control over the microstructure, mechanical properties, and fluid dynamics of biomaterials, thereby better simulating *in vivo* conditions and facilitating vascularization and functional maturation of COs. In summary, this review will systematically summarize recent advances in the use of biomaterials to promote vascularization of COs.

## Challenges in vascularization of COs

### Limitations in cell survival and nutrient supply within organoids

As is well known, the interactions between intercellular compartments (such as stroma, epithelium, and vasculature) collectively promote cross-talk between parenchymal cells and stromal cells through signal transduction, triggering overall function and facilitating organ maturation.^19^ Vessels in tissues are primarily involved in perfusion and the exchange of gases and metabolites, and the dependence of mature endothelial cells on glycolytic metabolism highlights the importance of vasculature in maintaining tissue metabolism and structural integrity.^20^ In the absence of a vascular network within organoids, cells are prone to hypoxia and nutrient deprivation, leading to reduced cardiomyocyte survival and impaired myocardial function. In contrast, angiogenesis helps improve the survival rate of COs under hypoxic and ischemic conditions.^21^ When COs are integrated with vascular organoids, this facilitates the vascularization of the COs, addressing challenges such as the formation of necrotic cores in large multicellular structures due to limited oxygen and nutrient supply. The incorporation of vascular cells also promotes the maturation of COs through paracrine signaling, making them more similar to adult organs.^22^

### The impact of lack of vascularization on organoid structure and function

Insufficient vascularization leads to increased heterogeneity among cardiomyocytes within organoids, thereby impairing myocardial contractile and conductive functions. This heterogeneity can be characterized along multiple dimensions, such as divergence in gene expression profiles and disparities in cardiomyocyte functional phenotypes. Single-cell transcriptomic analyses have revealed that COs lacking adequate vascularization exhibit marked variability in the expression of critical cardiac genes—including TNNT2, MYH6/MYH7, and NPPA—which are closely associated with sarcomere assembly and functional maturation.^4^ Additionally, genes involved in calcium handling—such as RYR2 and SERCA2a—are often underexpressed, resulting in an electrophysiological profile characteristic of immature cardiomyocytes.^8^ At the functional level, cardiomyocytes within poorly vascularized organoids display significant differences in contractile force, beat frequency, and action potential duration. These functional disparities can be quantified using techniques such as traction force microscopy and optical mapping.^23^ Furthermore, COs that lack proper vascularization are incapable of accurately recapitulating the pathophysiological processes underlying cardiac diseases, thereby limiting their utility in disease modeling and drug screening. Vascular formation plays a central role in several aspects: (1) promoting cardiomyocyte maturation—endothelial and perivascular cells interact with other cardiac-resident cell types (e.g., fibroblasts and cardiomyocytes) within organoids to advance maturation and enhance physiological function^24^; (2) enhancing contractile capacity—endothelial cells can secrete LAMA5 protein to regulate mature sarcomeric protein expression, thereby improving organoid contractility; concurrently, vascular cells drive extracellular matrix deposition via paracrine platelet-derived growth factor receptor beta (PDGFRβ) signaling, further strengthening contraction^24^; and (3) modulating disease responsiveness—during inflammation-induced diastolic dysfunction, vascular cell activity critically influences disease severity and promotes pathology via paracrine endothelin signaling.^24^ Thus, vascular cells not only support organoid maturation and function but also serve as key regulators in the organoid response to cardiac disease phenotypes, such as diastolic dysfunction.

### Differences between *in vitro* and *in vivo* vascularization

Organoids cultured *in vitro* cannot fully replicate the complex physiological conditions of *in vivo* environments, which limits the migration, proliferation, and differentiation of endothelial cells. The immaturity of COs due to insufficient vascularization exhibits features similar to those of the fetal human heart. These fetal characteristics make them more suitable for studying cardiac development and/or congenital heart disease, rather than adult cardiac (patho)physiology. Therefore, although reports have shown that physiological interventions, such as electrical stimulation, can promote the maturation of COs and engineered heart tissues (EHTs) to some extent, it remains challenging to apply these methods to model adult cardiac diseases, such as arrhythmias.^23^^,^^25^ Moreover, insufficient vascularization restricts the growth and function of COs, thereby limiting their application in translational research. Although the addition of endothelial cells (ECs) and/or angiogenic growth factors (such as VEGF-A) contributes to the formation of vascular networks in COs to some extent, the lack of true blood perfusion remains one of the main obstacles facing current models.^26^^,^^27^ Therefore, there is a need to develop biomaterials that can better simulate the *in vivo* environment to promote vascularization in organoids.

## The mechanisms of biomaterials in vascularization

### Physical properties of biomaterials

#### Porosity

The porosity of biomaterials plays a critical role in regulating cell growth within the scaffold, nutrient transport, and endothelial cell migration. High-porosity materials offer superior spatial support for angiogenesis. For instance, hydrogel foams with porosity levels around 75% can effectively facilitate molecular diffusion, cell migration, cellular recruitment, extracellular matrix remodeling, and neovascularization.^28^ High-porosity melt electrowriting (MEW) scaffolds promote the formation of dense microvascular networks, and the accompanying structural modifications significantly modulate angiogenic responses, enhancing blood perfusion in defect areas even in the absence of exogenous growth factors.^29^ Compared to low-porosity materials, high-porosity scaffolds allow for more efficient cell infiltration and active migration, thereby accelerating early-stage angiogenesis.^30^

Previous studies have indicated that, in the context of cardiovascular tissue engineering, scaffold pore sizes ranging from 40 to 100 μm are more favorable for the formation of vascular-like structures, making them particularly suitable for organoid systems that require neovascular support. Notably, pore sizes between 25 and 60 μm have been identified as optimal, as they balance cell integration with effective nutrient diffusion, thereby providing a more biomimetic microenvironment for COs.^31^ These design parameters offer important guidance for engineering physiologically relevant microenvironments in COs, ultimately enhancing their functional performance and long-term stability.

#### Elastic modulus

The mechanical properties of cardiac tissue play a pivotal role in regulating the behavior and function of both cardiomyocytes and endothelial cells. Adequate mechanical strength is essential for supporting endothelial cell survival.^32^ During the formation of vascular-like structures, fragile hydrogel matrices often undergo rapid remodeling and degradation, resulting in cell-mediated compression of the construct and the formation of dense but poorly interconnected capillary-like structures.^33^ Modulating the elastic modulus of biomaterials can direct endothelial cell differentiation and vascular network formation. For instance, a collagen-hyaluronic-acid (HA)-based hydrogel platform has demonstrated that tunable plasticity can be achieved through the combination of dynamic and covalent crosslinking strategies. Appropriate mechanical properties promote endothelial cell-mediated matrix remodeling, while a well-balanced matrix plasticity enhances both cell-matrix interactions and intercellular adhesion, thereby facilitating vascular assembly and invasion.^34^

The optimal elastic modulus range for supporting endothelial cell proliferation and angiogenesis is generally between 30 and 500 kPa. Within this range, materials with intermediate stiffness most closely mimic the mechanical properties of native tissues, avoiding the functional impairments associated with matrices that are either too soft or too stiff. This range is supported by experimental evidence from applications such as bioprinting and hydrogel-based organoid systems.^35^^,^^36^^,^^37^^,^^38^ Future research should aim to further optimize the modulus design for specific organoid systems to enhance angiogenic efficiency.

### Chemical properties of biomaterials

#### Surface chemical groups of materials

The chemical groups on the material surface have a significant impact on the adhesion and migration of endothelial cells, which is crucial in the design of biomaterials and endothelialization studies. Research has shown that different chemical groups can alter the interaction between cells and the material surface, thereby regulating cell behavior. In one study, surfaces with different chemical functional groups (such as CH_3_, NH_2_, COOH, and OH) were fabricated using self-assembled monolayers (SAMs) as a model system to investigate how these surfaces affect endothelial cell adhesion and migration.^39^ These chemical groups influence the surface’s chemical properties, such as hydrophilicity or hydrophobicity, and provide specific ligands that can either promote or inhibit cell adhesion. For example, surfaces with amino (NH_2_) groups generally promote cell adhesion because amino groups can form hydrogen bonds with ECM proteins, enhancing the interaction between cells and the surface. Yusuke et al. studied the adhesion behavior of human umbilical vein endothelial cells (HUVECs) on surfaces with different chemical groups, finding that amino and carboxyl groups provided the best conditions for cell growth.^40^Additionally, the chemical groups on the material surface can influence cell migration behavior. One study found that ECs cultured on 15-μm wide adhesive line patterns exhibited three distinct migration phenotypes: “running” cells that migrated continuously, “crawling” cells that migrated more slowly, and “anchored” cells that barely migrated.^41^ This indicates that by precisely controlling the surface chemical patterns, EC migration behavior can be modulated. In another study, a layer of methacrylated HA was first fixed on a poly(ε-caprolactone) (PCL) membrane, followed by dopamine coupling to introduce a dense EC-selective adhesion layer, thereby promoting selective adhesion and migration of ECs while inhibiting the adhesion and migration of smooth muscle cells (SMCs).^42^ This approach takes advantage of the different responses of various cell types to surface chemical signals, enabling precise control over EC and SMC adhesion and migration behaviors.

Furthermore, research has demonstrated that altering the chemical groups on the material surface can modulate EC migration behavior and associated signaling pathways. For example, introducing specific chemical groups on the material surface can activate integrin signaling pathways related to EC migration, thereby promoting EC migration.^39^ This suggests that by precisely controlling the chemical groups on the material surface, not only can EC adhesion and migration behavior be regulated but their associated cellular signaling pathways can also be influenced.

#### Bioactivity of materials

By incorporating ECM components, growth factors, or adhesion peptides into biomaterials, the proliferation and differentiation of ECs can be further promoted. Kong et al. developed a hybrid hydrogel composed of decellularized cardiac ECM and immunomodulatory glycopeptides for endogenous tissue regeneration following myocardial infarction (MI). This hydrogel constructs a niche that mimics the structure of natural ECM, attracting host cells for homing, controlling macrophage differentiation through glycopeptide units, and promoting EC proliferation via macrophage-endothelial cell crosstalk, thereby coordinating the intrinsic healing mechanisms of cardiac tissue regeneration.^43^ VEGF is a critical positive regulator of EC proliferation and vascular development.^44^ Gautrot et al. from University College London proposed a hydrogel based on 2-ethyl-2-oxazoline and 2-butene-2-oxazoline for enhancing angiogenesis. *In vitro* microfluidic experiments demonstrated that partially degradable hydrogel can upregulate vascular growth factors and improve angiogenesis.^45^ In a rat model of MI, implantation of hydrogel with mesenchymal stem cells (MSCs) in the epicardium helped restore cardiac function and structure while promoting new blood vessel formation. Wu et al. described another strategy involving the development of an injectable conductive alginate hydrogel containing polyaniline (PANI) and adeno-associated virus (AAV9-VEGF) for the treatment of MI.^46^ This hydrogel successfully transduced cardiomyocytes, leading to VEGF overexpression, which enhanced HUVEC proliferation, migration, and tube formation. Fan et al. created a microneedle patch made from graphene oxide and polyvinyl alcohol for controlled and sustained VEGF release.^47^ The microneedle patch significantly promoted neovascularization, reduced myocardial fibrosis, and restored heart function, demonstrating its potential in treating MI. A tissue-engineered vascular graft with high patency was constructed by combining heparin, cell adhesion peptides, and a carbon monoxide (CO) nanogenerator. Fan et al. prepared a CO nanogenerator based on covalent organic frameworks (COFs) and co-fixed it with LXW-7 cell adhesion peptides and heparin on decellularized blood vessels to construct a multifunctional SDTEVG. This SDTEVG not only prevents thrombosis and captures endothelial-forming cells (EFCs) to promote rapid endothelialization but also inhibits inflammation-mediated endothelial-mesenchymal transition (EndMT) through responsive CO release, maintaining endothelial homeostasis and preventing pathological intimal hyperplasia.^48^

### The role of biomaterials in promoting endothelial cell adhesion, migration, and differentiation

Biomaterials can promote the adhesion and migration of endothelial cells within organoids, thereby forming a three-dimensional vascular network by modulating their physicochemical properties. Yao et al. developed a dihydrolipoic-acid-modified sulfonated betaine-derived starch (SB-ST-D) hydrogel coating for polyethylene terephthalate (PET)-based blood-contacting devices. This coating facilitates the adhesion, proliferation, and migration of HUVECs through the formation of disulfide bonds, which holds potential for application in organoid vascularization.^49^

Natural hydrogels derived from decellularized extracellular matrix (dECM) from pig and human kidneys have been developed as scaffold materials that provide a more *in vivo*-like environment for stem cells. dECM hydrogels promote stem cell differentiation into kidney organoids and significantly enhance the formation of endogenous vascular components within the organoids. By combining kidney organoids with endothelial cell organoids, three-dimensional self-organized kidney-endothelial cell assembloids with vascular-like structures were generated, achieving vascularization of the organoids.^50^ This method not only enhanced the degree of vascularization in organoids but also improved the renal differentiation characteristics, making the organoids more closely resemble real kidney tissue.

**Figure 1:**
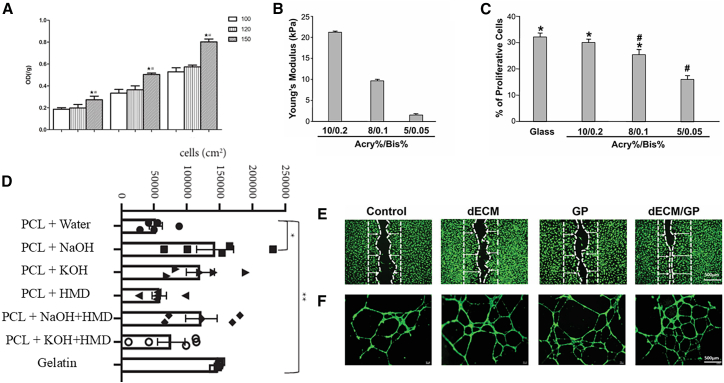
The influence of biomaterial properties on endothelial cell behaviors and angiogenesis (A) High porosity enhances cell adhesion and proliferation (n = 3; #, the difference attained a statistically significant increase compared to the 120 μm group, P < 0.05; the difference attained a statistically significant increase compared to the 100 μm group, P < 0.05. Error bars represent SEM).^51^ Reproduced from Figure 4,^51^ Sci. Rep., published under the Creative Commons Attribution 4.0 International Public License (CC BY 4.0; https://creativecommons.org/licenses/by/4.0/). (B and C) Materials with tissue-matching Young’s modulus boost HUVEC proliferation (∗ P < 0.05 when compared to hydrogel 5/0.05. # P < 0.05 when compared to glass. Error bars represent SEM).^52^ Reproduced from Figure S1,^52^ PLoS One, published under the Creative Commons Attribution License, which permits unrestricted use, distribution, and reproduction in any medium, provided the original author and source are credited. (D) Surface chemical groups regulate cell adhesion proteins, improving HUVEC adhesion and migration (Data are presented as mean±SEM. Statistical signifcance was tested with one-way ANOVA. ∗p < 0.05, ∗∗p < 0.01).^53^ Reproduced from Figure 7,^53^ J. Tissue Eng. Reg. Med., published under the Creative Commons Attribution License, which permits unrestricted use, distribution, and reproduction in any medium, provided the original work is properly cited. (E and F) Biomaterials promote angiogenesis by facilitating endothelial cell adhesion, migration, and differentiation (E, n = 5; F, n = 3; scale bar = 500 μm).^43^ Reproduced from Figure 4,^43^ Adv. Sci., published under the terms of the Creative Commons Attribution License, which permits use, distribution, and reproduction in any medium, provided the original work is properly cited.

Several factors significantly influence the process of angiogenesis, including the porosity of materials, elastic modulus, surface chemical groups, and their regulatory effects on endothelial cell adhesion, proliferation, migration, and differentiation. In this section, we summarize the key findings from various studies and present them visually in Figure 1. High-porosity materials significantly enhance cell adhesion and proliferation (Figure 1A). Materials with tissue-matching elastic modulus effectively promote HUVEC proliferation (Figures 1B and 1C). Surface chemical groups regulate adhesion proteins, improving HUVEC adhesion and migration (Figure 1D). Moreover, biomaterials further promote angiogenesis by enhancing endothelial cell adhesion, migration, and differentiation (Figures 1E and 1F).

## Common biomaterials and their applications in cardiac organoid vascularization

### Natural biomaterials

#### Collagen

Collagen is the primary structural protein in the extracellular matrix and is widely distributed in connective tissues such as the skin, bones, heart, and tendons.^54^ Due to its excellent biocompatibility and biodegradability, collagen has been extensively applied in cardiovascular tissue engineering and regenerative medicine.^55^ In ischemic conditions such as MI, collagen-based scaffolds or hydrogels are commonly used to deliver endothelial cells, growth factors, or extracellular vesicles to promote neovascularization and repair of ischemic tissues, thereby improving cardiac function.^56^^,^^57^ Type I and type IV collagens play crucial roles in the formation of vascular basement membranes and the regulation of endothelial cell behavior. Studies have shown that, in rat aortic models, type IV collagen at a concentration of 300 μg/mL can increase microvessel length by 119%, significantly enhancing endothelial cell migration, survival, and tube formation capabilities.^58^ In addition, collagen contributes to the regulation of cardiomyocyte electrophysiology, anti-apoptotic responses, and the maintenance of tissue architecture. Its degradation products—such as canstatin, tumstatin, and endostatin—have been shown to participate in the repair of myocardial ischemic injury by modulating calcium channels, attenuating oxidative stress, and regulating myofibroblast activity.^59^ Therefore, collagen not only serves as a structural support material but also exhibits multiple biological functions in the treatment of CVDs, highlighting its broad clinical application potential.

**Figure 2:**
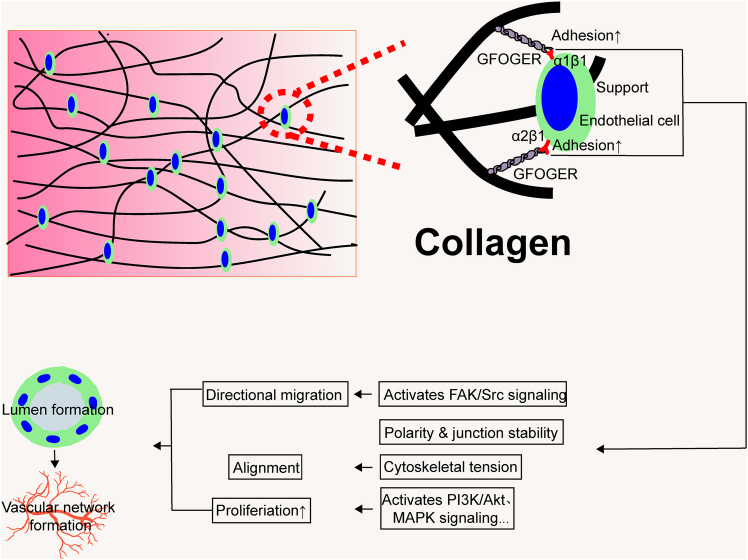
Schematic illustration of the role of collagen in promoting vascularization of cardiac organoids As a major component of the extracellular matrix, collagen facilitates the construction of vascular networks by providing adhesion sites, regulating cytoskeletal tension, and activating signaling pathways to promote endothelial cell adhesion, migration, and lumen formation.

In studies of cardiac organoid vascularization, collagen—one of the principal components of the ECM—has been widely employed to construct biomimetic organoid microenvironments due to its excellent biocompatibility and biodegradability. Collagen provides abundant adhesion sites for endothelial cells, facilitating their attachment and migration within three-dimensional matrices and thereby accelerating the formation of vascular networks.^60^ Beyond offering physical support, collagen also interacts with cells to generate traction forces via integrin-mediated focal adhesions and cytoskeletal tension, thereby influencing endothelial cell polarity and stabilizing lumen formation. In three-dimensional collagen matrices, endothelial cells form lumen structures through MT1-MMP-dependent proteolysis and cytoskeletal reorganization, highlighting collagen’s role in guiding cell morphology during angiogenesis.^61^ Through specific interactions with cell surface receptors, collagen activates multiple signaling pathways that regulate cell adhesion, migration, proliferation, and differentiation, playing a pivotal role in angiogenesis and tissue regeneration.^62^ This is attributed to collagen’s specific binding to integrins—a key family of transmembrane receptors linking the ECM to the cytoskeleton—via its GFOGER motif located in the triple-helical structure. This GFOGER-integrin-dependent adhesion mechanism not only strengthens endothelial cell anchorage to the matrix but also activates downstream signaling cascades that regulate cell migration, morphogenesis, and branching during angiogenesis.^63^ The multifaceted roles of collagen in vascular formation are illustrated in Figure 2.

Notably, although collagen exhibits outstanding advantages in promoting vascular network formation, it possesses several intrinsic limitations, including poor thermal stability, low solvent resistance, inadequate mechanical strength, and relatively slow biodegradation.^64^ These limitations hinder the precise control over its mechanical properties and degradation kinetics, which may compromise the stability of the vascular networks and the maturation of engineered tissues. Moreover, relying solely on collagen-based materials remains insufficient to fully replicate the complex biomechanical and biochemical microenvironment of myocardial tissue. Therefore, future research should focus more on composite strategies that integrate collagen with other natural or synthetic biomaterials. Such synergistic combinations are expected to further enhance the bioactivity, mechanical properties, and degradation behavior of the scaffolds, thereby facilitating the stable formation of functional vascular networks. Additionally, elucidating the molecular mechanisms underlying collagen-cell interactions and fine-tuning the bioactive sites and structural characteristics of collagen-based materials will be crucial for improving their clinical translation potential in the vascularization of COs.

#### Fibrin

Fibrin, as a natural biomaterial, plays an important role in tissue engineering and regenerative medicine. It not only possesses excellent biocompatibility and biodegradability but also features a nanofibrous structure that closely mimics the extracellular matrix, making it particularly well suited for tissue regeneration applications.^65^ Fibrin gel is formed through the polymerization of fibrinogen and thrombin derived from plasma. This naturally occurring biomaterial has been extensively studied due to its favorable biocompatibility, rapid biodegradation, and ease of fabrication, making it suitable for regenerating various tissues.^66^ Multiple studies have demonstrated that the injection of fibrin into the infarcted region of the rat’s left ventricle can significantly enhance the survival of transplanted cells, reduce infarct size, increase blood flow in the ischemic myocardium, and improve cardiac function.^67^^,^^68^

The three-dimensional structure of fibrin gel provides essential mechanical support for endothelial cells, facilitating vascular network formation. Its moldability allows it to be formed and solidified *in vivo*, making it a promising candidate for repairing tissue defects, particularly in vascular tissue engineering.^69^ The unique properties of fibrin gel permit tunable gelation time by adjusting polymerization parameters, resulting in soft yet physiologically stable matrices.^70^ Furthermore, fibrin-based biomaterials often outperform synthetic alternatives in mimicking the architecture and biochemical composition of native ECM, thereby enhancing regenerative outcomes.^71^ The nanofibrous structure, along with its tunable mechanical strength and degradation rate, underpins fibrin gel’s broad applicability in tissue engineering, especially in the context of promoting vascular regeneration.^72^

**Figure 3:**
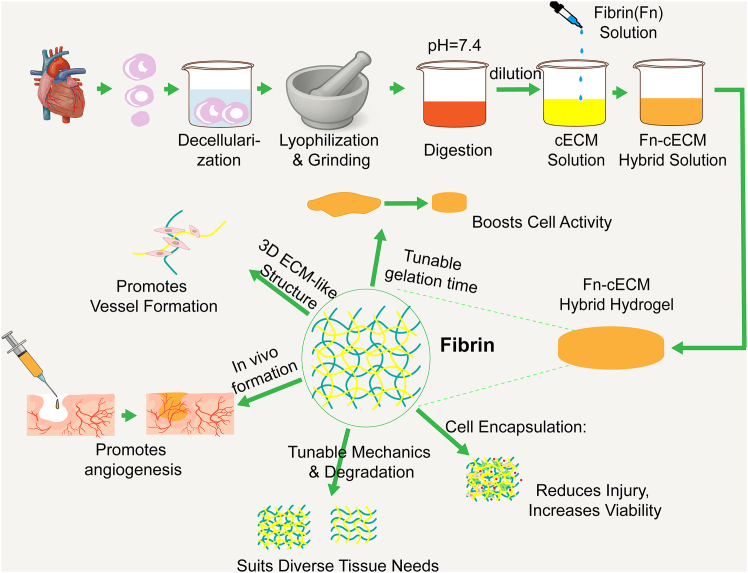
Schematic illustration of the preparation of fibrin-enriched cECM hydrogel and its mechanism in promoting angiogenesis Incorporation of fibrin into decellularized cardiac extracellular matrix (cECM) yields a composite hydrogel with tunable gelation time, mechanical properties, and degradability. This hydrogel enhances endothelial cell adhesion, proliferation, and vascular network formation, boosts the angiogenic potential of mesenchymal stem cells, and provides protection during delivery, thereby promoting tissue repair and vascular regeneration.

When incorporated into decellularized cardiac ECM (cECM), fibrin gives rise to a fibrin-enriched cECM hydrogel. This composite material has been shown to significantly promote the formation of capillary-like structures by HUVECs *in vitro* and to enhance mesenchymal stem cell sprouting and angiogenic activity.^73^ In vascular formation, fibrin gel functions not only as a carrier that shields cells from mechanical stress during delivery but also as a bioactive matrix that supports cell viability and promotes tissue regeneration.^74^ Additionally, fibrin gel has been applied in studies of implant vascularization strategies to help screen biomaterials for clinical translation.^75^ A typical fibrin hydrogel—the synthesis process of fibrin-enriched cECM hydrogel and its angiogenic mechanisms—is illustrated in Figure 3.

However, the application of fibrin in cardiac organoid vascularization still faces several challenges. On one hand, although fibrin hydrogels possess highly tunable mechanical properties and degradation rates, achieving precise modulation of these parameters to match the dynamic biomechanical environment of myocardial tissue remains a significant hurdle in practice. On the other hand, fibrin-based hydrogels alone may exhibit limited mechanical support and insufficient structural stability. Therefore, future studies may focus on developing composite materials by integrating fibrin with other natural or synthetic biomaterials to enhance mechanical robustness and biofunctionality, thereby better meeting the stringent requirements for scaffold materials in vascular network construction. Additionally, the molecular mechanisms governing fibrin-cell interactions require further elucidation, particularly regarding how nanofibrous architectures modulate cellular behavior and signal transduction. Such investigations will provide essential theoretical support for advancing the application and clinical translation of fibrin-based biomaterials in the vascularization of COs.

#### Hyaluronic acid

HA is a naturally occurring glycosaminoglycan widely distributed in the ECM of cartilage, connective tissues, the vascular system, and the heart.^76^ It plays a crucial regulatory role in various tissue remodeling processes, including embryonic development, wound healing, angiogenesis, and inflammatory responses.^76^^,^^77^ In CVD models, HA has also demonstrated promising reparative potential. In a rat model of MI, HA hydrogels were shown to improve left ventricular function, enhance collagen deposition, and reduce myocardial fibrosis.^78^ Furthermore, the degradation product oligomeric hyaluronic acid (o-HA; <10 disaccharide units) has been reported to activate the expression of *Ccl2* and *Cxcl5*, promote M2 macrophage polarization, suppress neutrophil-associated inflammation, and further activate MAPK and JAK/STAT signaling pathways, thereby accelerating cardiac functional recovery and improving compensatory cardiac remodeling.^79^ These findings highlight the multifaceted roles of HA in cardiac tissue repair, inflammatory regulation, underscoring its potential clinical utility as a therapeutic biomaterial for CVD.

**Figure 4:**
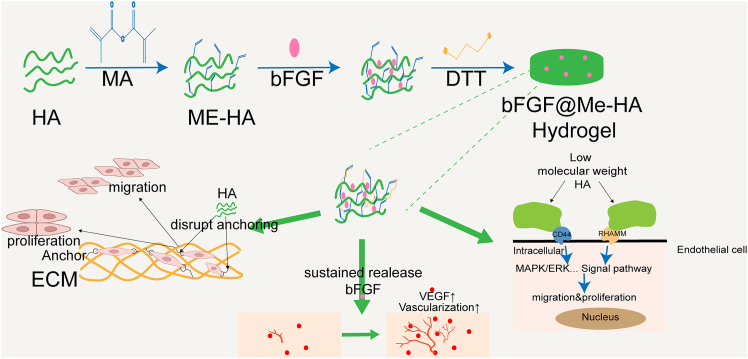
Schematic illustration of the synthesis process of bFGF@Me-HA hydrogel and its mechanism in promoting angiogenesis This hydrogel is fabricated by incorporating basic fibroblast growth factor (bFGF) into a methacrylated hyaluronic acid (Me-HA) network. The bFGF@Me-HA hydrogel enables efficient release of growth factors and synergistically activates multiple signaling pathways, thereby promoting the proliferation, migration, and differentiation of endothelial cells and vascular smooth muscle cells and enhancing both the quantity and maturity of neovessels.

HA is also a key regulator of cell proliferation, migration, and differentiation. Its presence helps maintain local tissue hydration, weakens the ECM’s anchoring effect on cells, and facilitates cellular motility through interactions with kinases involved in cytoskeletal dynamics. During early mitosis, the intracellular concentration of sodium hyaluronate increases significantly, then declines sharply as cells progress into the G1 phase. Elevated HA levels can stimulate the release of growth factors and influence intercellular communication by forming an ECM-like layer, thereby accelerating cell proliferation.^80^ The regulatory effects of HA are dependent on its molecular weight. Low-molecular-weight HA promotes cell proliferation and upregulates pro-inflammatory gene expression, whereas high-molecular-weight HA exerts the opposite effects, suppressing endothelial cell proliferation and migration while exhibiting anti-angiogenic properties.^81^^,^^82^ In the context of angiogenesis, HA also acts as a free ECM polymer that activates multi-level signaling cascades to promote smooth muscle cell differentiation, migration, and proliferation, contributing to vascular wall thickening.^83^ Moreover, HA can be readily chemically modified for multifunctionalization, crosslinked to form stable hydrogels, and engineered to possess tunable degradation rates and mechanical properties. When combined with angiogenic growth factors such as basic fibroblast growth factor (bFGF), HA-based hydrogels—for instance, bFGF@Me-HA—demonstrated a higher density and greater maturity of capillary structures during tissue regeneration compared to bFGF or Me-HA alone.^84^ This suggests that the combination of HA with pro-angiogenic factors more effectively promotes cell proliferation and neovascularization, making HA a critical component in enhancing vascularization within organoid systems. A typical example of such a growth-factor-loaded HA hydrogel—the synthesis process of the bFGF@Me-HA hydrogel and its pro-angiogenic mechanism—is illustrated in Figure 4.

In summary, HA has emerged as a prominent research focus in recent years for cardiac tissue engineering and organoid vascularization due to its excellent biocompatibility, tunable physicochemical properties, and synergistic effects with pro-angiogenic factors. Based on current research progress, it is anticipated that HA-based materials still face several challenges in the context of cardiac organoid vascularization. These challenges include the precise regulation of molecular weight distribution, degradation kinetics, and bioactivity, as well as the ability to better mimic native cECM within a three-dimensional microenvironment to support stable vascular structure formation. Furthermore, the integration of HA with other natural or synthetic materials, along with its incorporation into emerging biofabrication technologies such as 3D bioprinting, may unlock greater potential in future vascularization strategies and accelerate the clinical translation of COs.

#### Comparative analysis and application perspectives of natural biomaterials

In the field of cardiac organoid vascularization, collagen, fibrin, and HA represent three prototypical natural biomaterials, each possessing distinct characteristics and offering unique advantages in different application scenarios.

Collagen, known for its excellent biocompatibility and inherent cell adhesion motifs, is widely utilized to mimic the ECM, facilitating endothelial cell adhesion, migration, and vascular network formation. It is particularly suitable for constructing biomimetic organoid microenvironments and simulating the vascular basement membrane. However, collagen suffers from several inherent limitations, including poor thermal and solvent stability, low mechanical strength, and limited control over its degradation rate, which restrict its application in load-bearing or long-term support environments.^64^ Moreover, natural sources of collagen (e.g., bovine or porcine) exhibit substantial batch-to-batch variability, compromising reproducibility and clinical translation.

Fibrin offers high plasticity and *in situ* gelation capacity, with its gelation time and mechanical properties tunable via polymerization conditions. It can function both as a cell carrier and as a promoter of angiogenesis, making it well suited for injectable applications or composite hydrogel fabrication. Fibrin has shown promising results in the repair of MI sites. Nonetheless, its relatively fast degradation and low mechanical strength limit its use as a standalone long-term structural scaffold.^85^^,^^86^ Additionally, as fibrin is typically derived from blood products, concerns regarding batch variability and the risk of viral contamination must be addressed.

HA, owing to its molecular-weight-dependent bioactivity, offers favorable tissue affinity and the ability to modulate the immune microenvironment. It plays a critical role in regulating inflammation and promoting neovascularization, making it widely applicable in tissue repair and regeneration. HA is amenable to chemical modification, allowing for multifunctionalization and prolonged degradation, thus supporting fine-tuned control over vascularization and immune responses. However, high-molecular-weight HA can exhibit anti-angiogenic properties. Moreover, native HA has poor physical stability and is rapidly degraded by hyaluronidase, with an *in vivo* half-life ranging from hours to a few days. Without appropriate crosslinking strategies, it is challenging to maintain HA’s mechanical integrity and retention in tissue.^87^

In conclusion, although all three materials are pivotal in vascularization research, collagen is most suitable for providing biological adhesion support, fibrin for short-term tissue repair and cell delivery, and HA for immune modulation and development of multifunctional composite systems. The selection of an appropriate material should be tailored based on the specific structural and functional requirements of the target tissue.

### Synthetic biomaterials

#### Polyethylene glycol

Polyethylene glycol (PEG), as a synthetic polymer, has shown broad application prospects in the treatment of CVDs due to its excellent biocompatibility, tunable mechanical properties, and chemical modifiability. For example, a study developed a reactive oxygen species (ROS)-responsive hydrogel composed of PAMB-G-TK and 4-arm-PEG-SG, designed to deliver liposomes co-loaded with SS-31 and sphingosine-1-phosphate (S1P). This system effectively targets damaged mitochondria in cardiomyocytes, scavenges excessive ROS, and simultaneously promotes angiogenesis, resulting in significantly improved cardiac function following MI.^88^ In another study, direct injection of PEG hydrogel into the infarcted area revealed that even without carrying therapeutic cells, the hydrogel could mechanically support the myocardium, suppress left ventricular remodeling, and enhance cardiac function.^89^ Additionally, PEG hydrogels have been combined with human iPSC-derived cardiomyocytes (hiPSC-CMs) and pro-angiogenic erythropoietin (EPO) in MI models. The composite system significantly improved ejection fraction and alleviated cardiac remodeling.^90^ These findings collectively demonstrate that PEG hydrogels not only hold great potential for cardiac repair but also serve as efficient delivery platforms for various therapeutic cells and bioactive factors.

**Figure 5:**
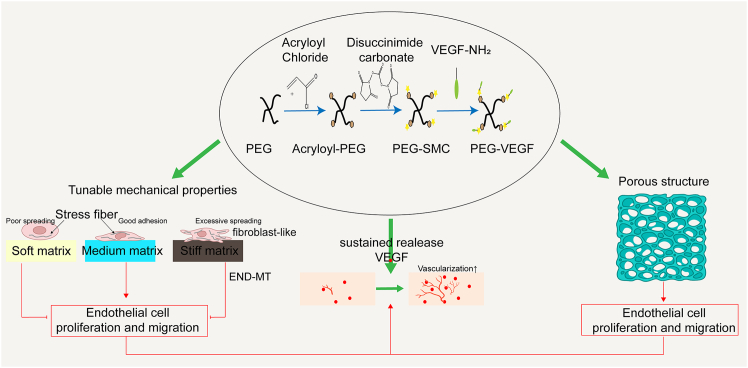
Schematic illustration of the synthesis process of PEG-VEGF hydrogel and its role in promoting angiogenesis This hydrogel is prepared by covalently conjugating VEGF within a PEG-based matrix. With tunable mechanical properties and a porous structure, the hydrogel enables sustained release of the growth factor, significantly enhancing endothelial cell proliferation, migration, and vessel formation.

As a versatile biomaterial, PEG hydrogels offer numerous advantages in promoting endothelial cell growth. By adjusting the concentration of crosslinkers or using different crosslinking methods, the mechanical properties of PEG hydrogels—such as elasticity and toughness—can be finely tuned, which is crucial for mimicking the biomechanical environment of native vasculature. For instance, studies have shown that substrate stiffness plays a vital role in regulating the self-renewal of muscle stem cells.^91^ Modifying the elastic modulus of PEG hydrogels can influence the growth preferences of different vascular cell types, enabling precise control over the angiogenesis process.^92^ Therefore, by varying the crosslinking density, PEG hydrogels with different stiffness levels can be tailored to suit the requirements of specific cell types. Moreover, the three-dimensional porous structure of PEG hydrogels provides a favorable microenvironment that facilitates cell migration and intercellular interactions. In the context of angiogenesis, PEG hydrogels can be further enhanced by covalently incorporating bioactive molecules such as VEGF.^88^ Studies have shown that covalently tethering VEGF within PEG hydrogels significantly improves the formation of tubular structures by endothelial cells, thereby promoting neovascularization.^93^^,^^94^ A typical example of such a cytokine-loaded PEG hydrogel—the synthesis process of PEG-VEGF hydrogel and its angiogenic mechanism—is illustrated in Figure 5.

In summary, PEG-based hydrogels have emerged as one of the key materials for constructing vascularized COs, owing to their high tunability, excellent cytocompatibility, and capacity to serve as carriers for a variety of bioactive molecules. Looking ahead, the application of PEG hydrogels in organoid vascularization can be further advanced in several directions. First, integrating PEG hydrogels with three-dimensional co-culture systems of iPSCs and vascular progenitor cells may enhance the spatial organization and functional maturation of intravascular networks within organoids. Second, employing bioprinting technologies to fabricate PEG hydrogel scaffolds with gradient stiffness or region-specific functionalities may improve the integration of heterogeneous cardiac cell populations and enhance vascularization efficiency within organoids. Although current challenges remain—such as the limited precision in the sustained release of bioactive molecules and the lack of intrinsic biological cues compared to natural matrices—PEG hydrogels, as a highly engineered material platform, hold significant promise for future applications in the development of functional vascular systems within COs.

#### Polylactic-co-glycolic acid

As a synthetic polymer widely used in tissue engineering and drug delivery, poly(lactic-co-glycolic acid) (PLGA) exhibits excellent biocompatibility, biodegradability, and tunable release properties, making it highly versatile for MI therapy. For instance, researchers have utilized PLGA nanoparticles to deliver VEGF, achieving sustained release for up to 30 days in a mouse MI model, which effectively promoted neovascularization in the ischemic region and enhanced myocardial tissue regeneration.^95^ In another study, PLGA microspheres were used for localized release of stromal-cell-derived factor 1α (SDF-1α), which enhanced stem cell chemotaxis toward injured myocardium, significantly improving cell recruitment efficiency and cardiac function.^96^ Moreover, composite PLGA patches have demonstrated remarkable regenerative capacity in animal models. A dual-mode electrospun fibrous scaffold composed of PLGA and collagen, loaded with bone-marrow-derived mesenchymal stem cells (BMSCs), was applied to infarcted myocardial tissue, leading to improved cell retention, suppression of left ventricular remodeling, and enhanced cardiac contractility.^97^ These preclinical findings highlight PLGA not only as an ideal vehicle for delivering drugs and growth factors but also as a promising scaffold for cardiac tissue engineering with great potential in cardiovascular therapy.

One of the key advantages of PLGA lies in its multifunctionality, particularly its tunable degradation rate, which can be precisely controlled by altering its composition and structure to meet specific clinical needs.^98^ This characteristic is especially critical in angiogenesis, as vascularization plays an essential role in tissue regeneration and repair. The degradation rate of PLGA can be adjusted by modifying the lactic acid-to-glycolic acid ratio and the polymer’s molecular weight, enabling synchronization with blood vessel formation and tissue growth.^98^ For example, different lactic acid-to-glycolic acid ratios (e.g., 50:50, 75:25, and100:0) can be used to tailor the degradation kinetics to specific therapeutic goals.^99^

Additionally, PLGA promotes angiogenesis through its degradation products—lactic acid and protons (H^+^). Lactic acid, taken up by cells via monocarboxylate transporters MCT1/2, is oxidized into pyruvate, which inhibits prolyl hydroxylase domain protein 2 (PHD2), stabilizing hypoxia-inducible factor 1α (HIF-1α) and activating nuclear factor κB (NF-κB).^100^^,^^101^^,^^102^ This cascade upregulates VEGF receptor 2 (VEGFR2) and interleukin-8 (IL-8) expression, thereby enhancing angiogenesis.^100^^,^^101^ Concurrently, the oxidation of lactic acid consumes NAD^+^, reducing ADP-ribosylation of proteins and further amplifying VEGF signaling to promote neovascularization and collagen deposition.^103^ Lactic acid can also activate the G-protein-coupled receptor GPR81, lowering intracellular cAMP levels and triggering β-arrestin-mediated noncanonical signaling pathways, which upregulate amphiregulin expression and further support angiogenesis.^104^^,^^105^ On the other hand, the H^+^ released during PLGA degradation creates a locally acidic microenvironment, which activates acid-sensing ion channels (TRPV1 and ASICs) and proton-sensitive GPCRs (OGR1, GPR4, and TDAG8).^106^^,^^107^^,^^108^ This activation leads to the upregulation of AP-1 and NF-κB pathways, promoting VEGF and IL-8 expression and synergistically driving angiogenesis.^109^^,^^110^^,^^111^ Thus, PLGA facilitates tissue repair and neovascularization through dual mechanisms involving lactic acid and protons.

**Figure 6:**
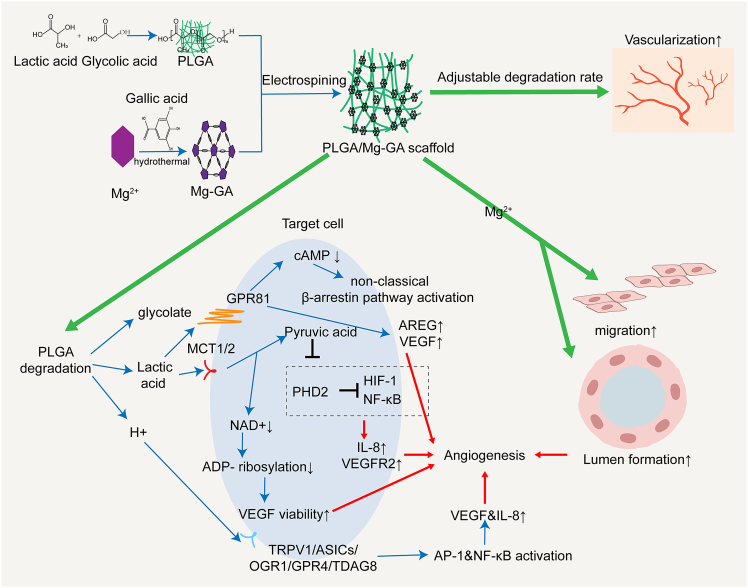
Schematic illustration of the synthesis process of the PLGA/Mg-GA composite scaffold and its mechanism in promoting angiogenesis This three-dimensional scaffold is fabricated by incorporating the metal-organic framework Mg-GA into a PLGA matrix via electrospinning, resulting in a structure with tunable degradation rates. As PLGA degrades, it releases lactic acid and H^+^, which enhance HIF-1 and natural factor κB (NF-κB) activity by modulating multiple molecular pathways, including GPR81 signaling, PHD2 inhibition, and NAD^+^ depletion. This upregulates VEGF and IL-8 expression, synergistically promoting endothelial cell proliferation, migration, and neovascularization. Meanwhile, the sustained release of Mg^2+^ facilitates endothelial cell adhesion, migration, and lumen formation, while GA contributes antioxidant and anti-inflammatory effects, improving the local microenvironment and reducing inflammation. The combined actions of these components coordinately regulate endothelial cell function and the local physiological milieu, significantly enhancing the angiogenic and tissue repair potential of the PLGA scaffold.

Furthermore, the incorporation of metal-organic frameworks (MOFs) into PLGA has yielded functional composite scaffolds. A representative example is the PLGA/Mg-GA composite scaffold, where the sustained release of Mg^2+^ ions enhance endothelial cell adhesion, migration, and tube formation. Gallic acid (GA), a natural polyphenol compound, provides antioxidant and anti-inflammatory effects, suppressing local inflammation and improving the ischemic-hypoxic microenvironment, which collectively supports endothelial cell proliferation and functionality. The synergistic action of Mg^2+^ and GA modulates both endothelial cell behavior and the local physiological environment, significantly enhancing the angiogenic potential of PLGA-based materials.^112^A representative example of a PLGA-based scaffold—the synthesis and angiogenic mechanism of the PLGA/Mg-GA composite scaffold—is illustrated in Figure 6.

In summary, as a highly engineered and tunable synthetic polymer, PLGA not only provides stable support for tissue repair and drug delivery but also exhibits great potential in organoid vascularization due to its unique degradation mechanisms and multi-pathway pro-angiogenic effects. Moving forward, the application of PLGA in cardiac organoid construction can be expanded in several key directions. First, by leveraging its adjustable monomer ratio, PLGA can be tailored to precisely match the vascularization rate and mechanical support required at different developmental stages of COs, enabling dynamic microenvironmental simulation. Second, combining PLGA with angiogenic factors, extracellular vesicles, or bioactive small molecules to form multifunctional composite systems may enhance the spatial integrity and functional maturation of vascular networks. Additionally, the integration of PLGA with advanced technologies such as microfluidics and 3D bioprinting holds promise for the precise fabrication and functional modulation of vascular conduits. Nonetheless, long-term immune responses, comprehensive safety evaluations, and electrical integration with myocardial tissues remain to be fully addressed in organoid systems. Therefore, through continued optimization of material properties and biointerface design, PLGA offers a scalable and innovative platform for advancing vascularized organoid engineering.

#### Polycaprolactone

Polycaprolactone (PCL) is a biodegradable polyester that has attracted significant attention due to its excellent biocompatibility, the Food and Drug Administration (FDA)-approved clinical applications, biodegradability, and low immunogenicity.^113^ These attributes make PCL a valuable material in tissue engineering, particularly for cardiac tissue regeneration. PCL scaffolds can be engineered to mimic the structural and mechanical characteristics of cardiac tissue by adjusting fiber orientation and porosity, making them especially suitable for cardiac organoid development.^114^ In addition, PCL scaffolds degrade slowly *in vivo*, providing ample time for cellular infiltration and the establishment of vascular networks.^115^ Electrospun PCL nanofiber scaffolds doped with 1% graphene have demonstrated significantly enhanced mechanical strength and electrical conductivity. These composite scaffolds also markedly improve the adhesion and proliferation of both cardiac progenitor cells and endothelial cells. Such scaffolds not only provide a myocardial-like microenvironment but also show promise as bioactive substrates for cardiac injury repair,^116^ underscoring their potential clinical utility. In cardiac tissue engineering, the robust mechanical properties and anisotropic structure of PCL scaffolds are essential for replicating the native architecture of the heart. Studies have demonstrated that PCL scaffolds effectively support cardiomyocyte adhesion, proliferation, and differentiation—processes that are critical for myocardial repair and functional regeneration.^117^

In the context of angiogenesis, PCL’s material versatility allows it to be blended with other biomaterials to form composite scaffolds that optimize vascularization and improve cell proliferation and tissue regeneration efficiency.^118^ The inherent biocompatibility and biodegradability of PCL further enhance its value in medical applications, particularly in tissue engineering and controlled drug delivery.^119^ The degradation rate of PCL can be modulated by altering its composition and structural parameters, allowing for customized designs to meet specific tissue repair needs.^120^ For example, PCL-PLA (polylactic acid) composites fabricated via 3D printing have been extensively studied and reviewed for applications in bone, cardiac, neural, vascular, and skin tissue engineering.^120^

**Figure 7:**
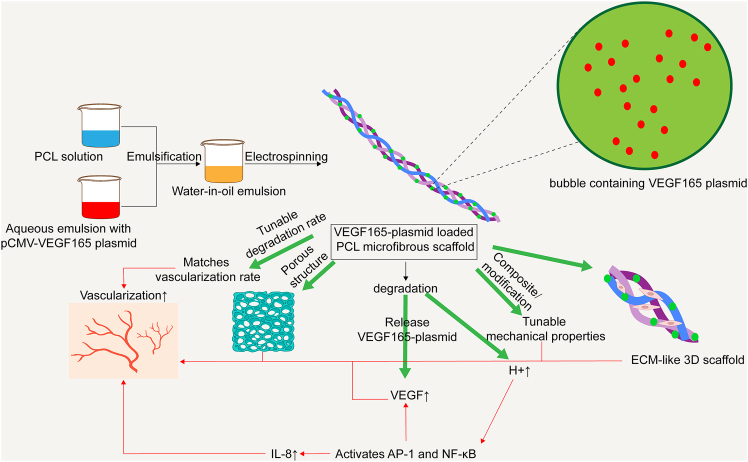
Schematic illustration of the synthesis process of the VEGF165 plasmid-loaded PCL microfiber scaffold and its mechanism in promoting angiogenesis This scaffold is fabricated by mixing an aqueous emulsion containing the pCMV-VEGF165 plasmid with a PCL organic solution, followed by emulsification and electrospinning to form a porous three-dimensional structure with a tunable degradation rate. During scaffold degradation, the embedded VEGF165 plasmid is gradually released, promoting VEGF and IL-8 expression and activating angiogenesis-related signaling pathways such as AP-1 and NF-κB. This significantly enhances endothelial cell proliferation, migration, and neovessel formation. Additionally, the angiogenic performance of the PCL scaffold can be further optimized by combining it with other materials or tuning its mechanical properties, enabling adaptation to various tissue repair and regeneration needs.

PCL scaffold vascularization can also be optimized through various strategies. For instance, combining PCL microfibrous scaffolds with pCMV-VEGF165 plasmid delivery has been shown to enhance angiogenesis in rat models, while also influencing scaffold degradation rates.^115^ Additionally, adjusting the ratio of PCL to chitosan (CTS) improves scaffold adaptability to accommodate both rigid and soft tissue defects.^118^ A representative example of such a scaffold—the synthesis process of the VEGF165-plasmid-loaded PCL microfibrous scaffold and its angiogenic mechanism—is illustrated in Figure 7.

Given the outstanding advantages of PCL in mechanical properties, degradation behavior, and structural plasticity, its application in the vascularization of COs holds significant promise. By constructing PCL scaffolds with anisotropic architectures mimicking native myocardium, it is possible to provide physical guidance for the aligned organization of vascular and cardiac cells within organoids, thereby facilitating the spatially ordered formation of functional vascular networks. Moreover, the excellent processability of PCL enables its integration with various bioactive factors, gene delivery systems, or conductive nanomaterials to further enhance its performance in angiogenesis, electrical signal conduction, and tissue integration. Future research may focus on the development of multimodal PCL-based composite scaffolds—for instance, incorporating extracellular vesicles or stimuli-responsive molecules—to improve vascular remodeling in dynamic microenvironments. Additionally, combining PCL with matrix-mimicking biomaterials could enhance endothelial phenotype maintenance and overall metabolic support within organoids. Although PCL has already demonstrated a solid foundation for clinical translation, optimizing the balance between its degradation rate and the tissue maturation process remains a key challenge to ensure long-term structural and bioactive support during organoid culture.

#### Comparative analysis and application perspectives of synthetic biomaterials

PEG, PLGA, and PCL are three widely used synthetic biomaterials, each possessing unique advantages for vascularization in COs. PEG hydrogels exhibit excellent biocompatibility and tunable mechanical properties, and they are easily modified, making them suitable for the delivery of various cell types and bioactive factors. Their three-dimensional porous structures provide a favorable microenvironment for cell migration and angiogenesis by modulating crosslinking density to meet specific cellular needs. However, PEG lacks inherent bioactivity and typically requires the incorporation of biological molecules (e.g., VEGF) to enhance its pro-angiogenic capacity. Although PEG is considered biocompatible, its limited biodegradability raises concerns for *in vivo* applications, as prolonged retention may lead to chronic inflammation or, in some cases, renal burden and hypersensitivity due to high-molecular-weight PEG accumulation.^121^

PLGA is well known for its favorable biodegradability and controlled drug/growth factor release properties. By adjusting the monomer ratio, the degradation rate can be precisely modulated to align with different tissue repair timelines. Furthermore, its degradation products can activate angiogenic signaling through multiple pathways, making PLGA an ideal material for drug delivery and scaffold systems. However, PLGA degradation may cause localized acidification, potentially irritating surrounding tissues, which necessitates optimized design strategies to mitigate adverse effects. PCL, on the other hand, offers superior mechanical strength, a slower degradation profile, and good biocompatibility, making it especially suitable for long-term support in cardiac and other mechanically demanding tissue engineering applications. PCL scaffolds can be finely structured via electrospinning or 3D printing, supporting applications in myocardial repair and vascular engineering. Nevertheless, due to its slow degradation and limited bioactivity, PCL is often combined with other materials or bioactive agents to enhance tissue regeneration and vascularization.^122^

In summary, PEG is ideal for cell delivery and microenvironment modulation, PLGA is suitable for tunable degradation and drug delivery applications, and PCL provides mechanical robustness for long-term support. The optimal strategy for constructing vascularized COs often involves the combinatorial use of these synthetic materials with natural polymers, growth factors, or other bioactive components to achieve synergistic effects and improve vascularization outcomes.

### Composite biomaterials

#### Gelatin methacryloyl hydrogel

Gelatin methacryloyl (GelMA), known for its excellent biocompatibility and photo-crosslinking properties, has been widely used in cardiac tissue engineering. In a murine model of MI, researchers applied GelMA hydrogel to the epicardial surface, resulting in significantly improved survival rates (89% vs. 50%), enhanced left ventricular systolic function (37% vs. 26%), and reduced scar area (6% vs. 22%) compared to untreated controls.^123^ Furthermore, conductive GelMA/rGO hydrogels have been used to deliver umbilical-cord-derived mesenchymal stem cells (UCMSCs), promoting *in vivo* cell survival, upregulating the expression of cardiac markers such as cTnI and Cx43, and improving ejection fraction and myocardial repair, thus demonstrating strong therapeutic potential in stem-cell-based cardiac therapies.^124^

GelMA combines the biological functionality of gelatin with the processability of photo-crosslinking, making it an ideal biomaterial for constructing biomimetic vascular networks, especially when integrated with 3D printing technologies. As a photopolymerizable hydrogel derived from gelatin, GelMA exhibits high biocompatibility and supports cell adhesion, proliferation, and differentiation.^125^^,^^126^ It also contains intrinsic cell-binding motifs, such as the RGD (arginine-glycine-aspartic acid) sequence, which facilitates robust cell attachment and growth.^127^ The photo-crosslinkable nature of GelMA enables precise spatial control during fabrication via 3D bioprinting, allowing for the design of complex vascular architectures.^128^^,^^129^ Through controlled printing parameters, GelMA hydrogels can be fabricated with tailored pore structures and mechanical properties that closely mimic the biomechanical and transport functions of natural vascular networks—key factors for supporting tissue vascularization in engineered constructs.

**Figure 8:**
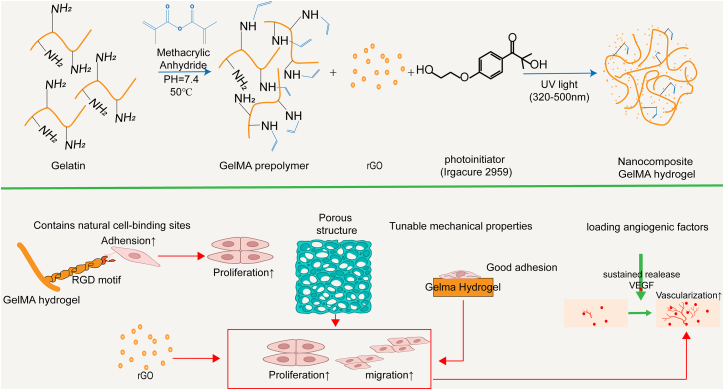
Schematic illustration of the synthesis process of the nanocomposite GelMA hydrogel and its mechanism in promoting angiogenesis This hydrogel is fabricated by photo-crosslinking methacrylated gelatin (GelMA) with nanomaterials such as reduced graphene oxide (rGO) in the presence of a photoinitiator under UV light. GelMA contains natural cell-binding motifs (e.g., RGD sequences), which significantly enhance cell adhesion and proliferation, while its porous structure and tunable mechanical properties provide an optimal microenvironment for endothelial cell growth and migration. The incorporation of rGO further boosts the bioactivity of the hydrogel. Additionally, GelMA can be loaded with angiogenic factors (e.g., VEGF) to enable sustained release and upregulation of VEGF and angiogenesis-related signaling, markedly enhancing neovascularization and tissue repair capacity.

Moreover, GelMA hydrogels can be functionally enhanced by incorporating bioactive components such as growth factors, cells, or nanoparticles to further promote angiogenesis and tissue regeneration.^130^ For example, culturing HUVECs expressing VEGF on GelMA-based microgels and implanting them into ischemic tissue has been shown to effectively induce neovascularization and restore blood perfusion.^130^ A representative example of a GelMA-based hydrogel—the synthesis and angiogenic mechanism of the nanocomposite GelMA hydrogel—is illustrated in Figure 8.

In summary, GelMA holds significant research and application value in constructing vascularized microenvironments for COs due to its favorable bioactivity, tunability, and high patterning precision. In future applications within cardiac organoid engineering, GelMA can be integrated with advanced multiscale fabrication technologies—such as 3D bioprinting and microfluidic systems—to better mimic the spatial architecture and mechanical features of native vascular networks. Furthermore, GelMA’s excellent biocompatibility and adjustable properties provide a versatile platform for delivering pro-angiogenic factors, extracellular vesicles, or ECM components. By dynamically tuning its mechanical characteristics and degradation rate, GelMA-based systems may more accurately replicate the native cardiac microenvironment, thereby enhancing vascularization efficiency and long-term functional stability. Nevertheless, systematic studies are still needed to elucidate GelMA’s hydrolytic degradation behavior during prolonged culture, its mechanical stability, and the synergistic interactions with other cardiac organoid matrix components, in order to advance its clinical translation for organoid vascularization.

#### PCL-PLGA composite scaffold

PCL-PLGA composite scaffolds combine the excellent mechanical strength of PCL with the favorable biodegradability of PLGA, making them a widely used and tunable polymer system for soft tissue engineering applications. By adjusting the component ratio, both the mechanical properties and degradation behavior of the scaffold can be finely controlled. For instance, increasing the PCL content enhances scaffold elasticity and porosity, while the incorporation of PLGA allows for better control over degradation rates, better aligning with the dynamic remodeling needs of myocardial tissue during repair.^131^^,^^132^ However, the acidic microenvironment resulting from the rapid degradation of PLGA may negatively impact the regenerative effects of PCL. To address this limitation, researchers have introduced bioactive ceramics such as β-tricalcium phosphate (β-TCP) or hydroxyapatite into the composite design to buffer local pH levels, reduce inflammation, and improve scaffold performance in both bone and myocardial regeneration contexts.^133^ In summary, PCL-PLGA composite scaffolds offer tunable mechanical and degradation profiles, presenting multifunctional potential for myocardial tissue engineering. Nonetheless, their direct application in cardiac regeneration remains to be further validated *in vivo*.

PCL contributes sufficient mechanical support, ensuring scaffold stability *in vivo*, while PLGA allows for controlled degradation, creating space and time for tissue ingrowth and neovascularization. These synergistic properties make the PCL-PLGA composite scaffold an ideal platform to support the growth and vascularization of COs.^134^ PCL-PLGA scaffolds can be engineered into biomimetic three-dimensional structures using various micro- and nanoscale fabrication techniques. These scaffolds promote endothelial cell adhesion and migration—key steps in vascular network formation. Common fabrication methods include electrospinning (ES) and solvent casting (SC)/particulate leaching, both of which enable precise control over pore architecture and mechanical strength.^135^ For example, 3D-printed PCL-PLGA scaffolds with a helical configuration have been shown to enhance mass transport compared to traditional cylindrical structures, thereby improving their functionality in tissue engineering applications.^136^

**Figure 9:**
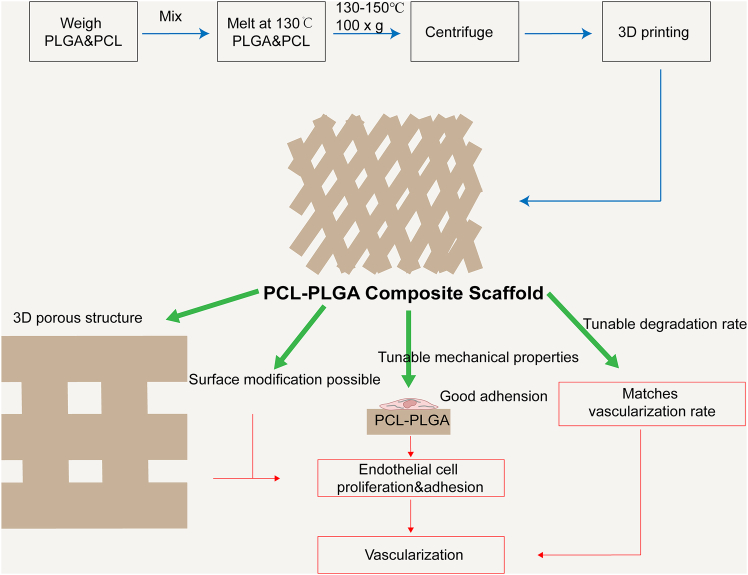
Schematic illustration of the synthesis process of the PCL-PLGA composite scaffold and its mechanism in promoting angiogenesis This scaffold is fabricated by weighing and mixing PLGA and PCL, followed by processes such as melting, centrifugation, and 3D printing to form a biomimetic three-dimensional porous structure. PCL provides excellent mechanical strength, while PLGA enables a tunable degradation rate, ensuring structural stability and biodegradability. By adjusting the PCL-to-PLGA ratio, the scaffold’s degradation and angiogenesis rates can be precisely matched to tissue repair needs. Surface modification can further enhance cell adhesion and biocompatibility. This composite scaffold supports endothelial cell proliferation, adhesion, and migration, significantly promoting neovascularization and offering an ideal platform for tissue engineering applications such as cardiac organoids.

Furthermore, the degradation kinetics of PCL-PLGA composites can be modulated by varying the PCL-to-PLGA ratio. Studies have demonstrated that scaffolds with a 0.5:0.5 PCL/PLGA ratio exhibit optimal *in vitro* biological responses, including stable nanofiber morphology and uniform molecular release, contributing to better control over tissue regeneration processes.^134^^,^^137^ Surface modification offers another strategy to further enhance scaffold biocompatibility and cellular interactions. For example, the incorporation of heparin-dopamine (Hep-DOPA) conjugates has been shown to significantly improve scaffold hydrophilicity, thereby promoting the adhesion and proliferation of human periodontal ligament stem cells (hPDLSCs).^134^ A representative example of such a scaffold—the synthesis process of the PCL-PLGA composite scaffold and its pro-angiogenic mechanism—is illustrated in Figure 9.

Overall, PCL-PLGA composite scaffolds exhibit promising potential in cardiac organoid vascularization owing to their tunable mechanical properties and degradation behavior. This type of scaffold leverages the mechanical stability of PCL and the controllable degradability of PLGA, offering dynamic structural support for COs. Such scaffolds maintain organoid integrity during culture while gradually degrading to provide space for neovascularization and extracellular matrix remodeling. Moreover, advanced micro- and nanoscale fabrication techniques, such as 3D printing, enable precise control over the scaffold’s architecture, allowing the recreation of vascular alignment patterns seen in native cardiac microenvironments. Future research should focus on optimizing the PCL-to-PLGA ratio and fine-tuning the scaffold’s microstructure, as well as investigating synergistic applications with pro-angiogenic factors, extracellular vesicles, and other bioactive agents to enhance overall vascularization performance. In addition, the integration of multiscale mechanical regulation and real-time imaging technologies could help elucidate the scaffold’s dynamic degradation mechanisms and its temporal influence on angiogenesis, ultimately supporting the development of physiologically relevant vascularized COs.

#### Gelatin-chitosan composite hydrogel

Gelatin-chitosan (Gel-CS) composite hydrogels have been demonstrated to provide a flexible and bioactive three-dimensional scaffold in myocardial injury environments. Researchers have developed a multilayered composite cardiac patch, in which a mechanically robust and surgically operable PCL core is encapsulated by a Gel-CS hydrogel shell to enhance cellular adhesion. This scaffold mimics the pore size and mechanical properties of native myocardium and supports the viability of neonatal rat ventricular myocytes (NRVMs), promoting their migration and spontaneous beating within the construct. It also exhibits favorable biodegradability and mechanical support, making it suitable for the repair of congenital heart defects.^138^ In another study, a decellularized porcine myocardial matrix was combined with chitosan to create a three-dimensional scaffold for right ventricular outflow tract (RVOT) replacement. The composite scaffold maintained cell viability, enhanced cardiomyocyte retention, and improved electrophysiological function, significantly upregulating α-myosin heavy chain and connexin-43 (Cx43) expression, as well as improving conduction velocity and contractile force. These findings highlight its potential as a biodegradable cardiac patch.^139^ These results collectively suggest that Gel-CS composite hydrogels, as soft yet bioactive scaffold materials, can provide both structural support and a regenerative microenvironment for injured myocardium, showing strong potential for applications in cardiac regeneration therapy.

**Figure 10:**
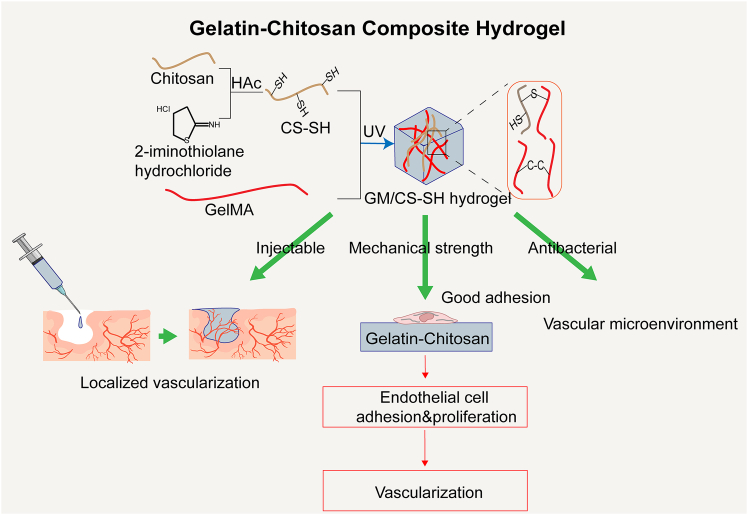
Schematic illustration of the synthesis process of the GM/CS-SH composite hydrogel and its mechanism in promoting angiogenesis This hydrogel is prepared by combining chitosan and gelatin through thiolation modification and UV-induced crosslinking, resulting in a material with excellent injectability, mechanical strength, and antibacterial properties. Its three-dimensional porous structure and biocompatibility provide an ideal microenvironment for vascularization, supporting endothelial cell adhesion and proliferation and promoting neovessel formation. Chitosan contributes to the hydrogel’s mechanical strength and antimicrobial activity, while gelatin enhances cell adhesion and bioactivity. The synergistic effects of both components effectively facilitate vascularization in engineered tissues such as cardiac organoids.

In terms of promoting vascularization, a composite scaffold composed of human cECM, chitosan, and gelatin—referred to as the cECM-CG scaffold—has been shown to effectively support the adhesion, growth, and endothelial differentiation of CD34^+^ endothelial progenitor cells (EPCs). This scaffold significantly upregulated vascular-related marker expression and induced tubular structure formation.^140^ Additionally, chitosan’s inherent antimicrobial properties impart the Gel-CS hydrogel with resistance to microbial invasion. This has led to the development of antibiotic-free wound dressings with notable antimicrobial efficacy and good biocompatibility.^141^ The antimicrobial nature of the Gel-CS hydrogel is especially advantageous for maintaining a sterile environment during the *in vitro* culture of COs. Moreover, chitosan contributes substantial mechanical strength to the composite, ensuring structural stability and resilience against external forces. For example, a hydrogel consisting of 50% gelatin and 50% chitosan exhibits a compressive modulus (∼15 kPa) comparable to that of native soft tissue, indicating the composite’s mechanical suitability for tissue engineering applications.^138^ Furthermore, a study by Samiei demonstrated that Gel-CS hydrogels possess excellent injectability, making them highly convenient for applications in organoid vascularization.^142^ A representative example of such a hydrogel—the synthesis process and angiogenic function of the GM/CS-SH composite hydrogel—is illustrated in Figure 10.

Given the combined advantages of mechanical properties, bioactivity, and structural tunability, Gel-CS composite hydrogels hold significant application potential in the vascularization of COs. On one hand, the tunable component ratio allows these hydrogels to mimic the mechanical environment of native myocardial tissue, thereby providing a favorable scaffold for endothelial cell adhesion, migration, and functional expression. Moreover, their excellent injectability enables precise localization and controlled release within microscale organoid systems. On the other hand, the natural origin, biodegradability, and intrinsic antimicrobial properties of Gel-CS hydrogels contribute to the maintenance of a sterile microenvironment during long-term culture or implantation simulations, thereby improving the survival and stability of vascular networks. Future research could further explore the compatibility of Gel-CS hydrogels with various cardiac organoid cell types (e.g., endothelial progenitor cells, cardiac progenitor cells), as well as their integration into microenvironment-responsive material systems. In combination with advanced techniques such as microfluidics and 3D bioprinting, these hydrogels may support the construction of vascular microstructures that closely resemble physiological conditions, offering a feasible material strategy for the development of functionally mature COs.

**Figure 11:**
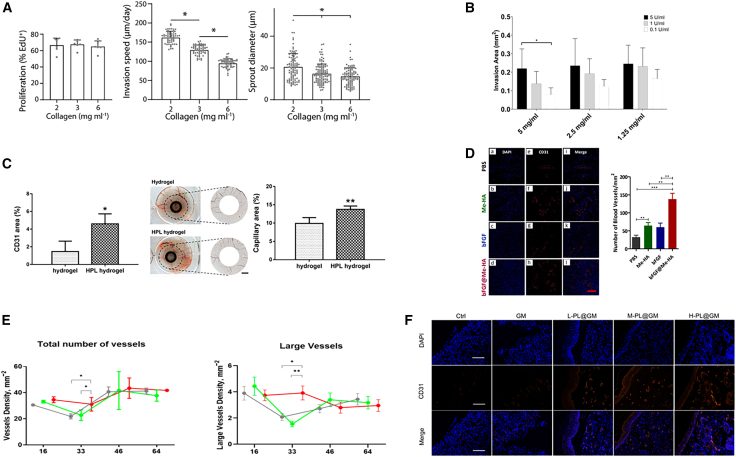
Diverse biomaterial scaffolds for promoting angiogenesis (A) Collagen matrix proteins at specific concentrations polymerize into fibrous scaffolds suitable for endothelial cell migration and invasion (All data presentedas mean ± SD; ∗ indicate sastatistically significant comparison with P < 0.05 (one-way ANOVA)).^143^ Reproduced from Figure 3,^143^ Front. Bioeng. Biotechnol., published under the terms of the Creative Commons Attribution License (CC BY). The use, distribution, or reproduction in other forums is permitted, provided the original author(s) and the copyright owner(s) are credited and that the original publication in this journal is cited, in accordance with accepted academic practice. (B) Fibrin-based gels promote angiogenesis (The asterisk symbol in the plot represents ∗ p < 0.05. The error bars represent the Standard Deviation. n = 5).^144^ Reproduced from Figure 6,^144^ J. Funct. Biomater., published under the terms and conditions of the Creative Commons Attribution (CC BY) license (https://creativecommons.org/licenses/by/4.0/). (C) Gelatin-chitosan hydrogels influencing vascularization (∗p < 0.05, ∗∗p < 0.01, scale bar = 3 mm).^145^ Reproduced from Figure 7,^145^ Int. J. Mol. Sci., published under the terms and conditions of the Creative Commons Attribution (CC BY) license (https://creativecommons.org/licenses/by/4.0/). (D) HA hydrogels facilitate angiogenesis (∗∗p < 0.01, ∗∗∗p < 0.001, scale bar = 200 μm).^84^ Reproduced from Figure 8,^84^ ACS Appl. Bio Mater.; permission to use material from this article has been obtained from the publisher (American Chemical Society). All rights reserved by the original publisher. (E) PCL scaffold (HCG group) enhances angiogenesis (Data presented as mean ± 95% CI. LCGroup-low-concentration group, HCGroup high-concentration group. Note: ∗p < 0.05, ∗∗ p <0.01).^115^ Reproduced from Figure 2,^115^ Int. J. Mol. Sci., published under the terms and conditions of the Creative Commons Attribution (CC BY) license (https://creativecommons.org/licenses/by/4.0/). (F) GelMA hydrogels with varying platelet lysate (PL) concentrations promote angiogenesis to different extents (scale bar = 200 μm).^146^ Reproduced from Figure 5,^146^ Sci. Rep., published under the terms and conditions of the Creative Commons Attribution (CC BY) license (https://creativecommons.org/licenses/by/4.0/).

Various biomaterials play pivotal roles in promoting angiogenesis by providing a supportive microenvironment for endothelial cells. Figure 11 visually summarizes these effects: collagen matrix proteins polymerize into fibrous scaffolds at specific concentrations, supporting endothelial cell migration and invasion (Figure 11A); fibrin-based hydrogels promote angiogenesis (Figure 11B); Gel-CS hydrogels influencing vascularization (Figure 11C). Additionally, HÀ hydrogels facilitate angiogenesis (Figure 11D). PCL hydrogels in the HCG group significantly enhance angiogenesis (Figure 11E), and GelMA hydrogels with varying PL concentrations promote angiogenesis to different extents (Figure 11F). These findings highlight the versatility of biomaterials in modulating the angiogenic process.

**Figure 12:**
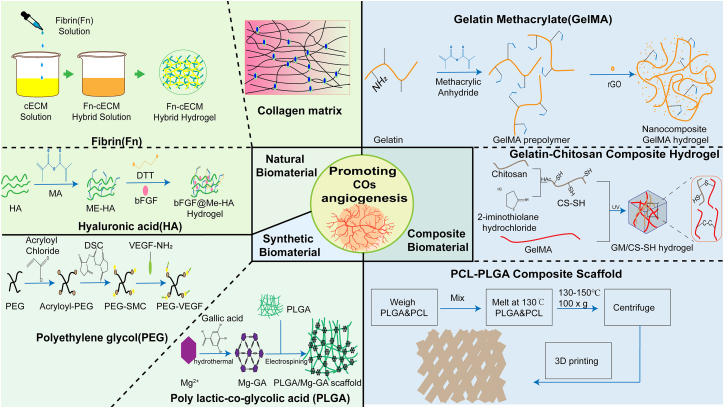
Schematic representation of the synthesis processes and key components involved in the development of various biomaterials, including collagen matrices, fibrin-based hydrogels, PEG hydrogels, HA hydrogels, gelatin-chitosan hydrogels, GelMA hydrogels with rGO, and PLGA scaffolds This figure highlights the chemical modifications and fabrication strategies that contribute to the creation of biomaterials designed to support angiogenesis.

The synthesis and structural characteristics of these biomaterials are critical for their biological functions in promoting angiogenesis. To provide a clearer understanding of the fabrication processes and material properties, a schematic representation summarizing the synthesis of these materials is shown in Figure 12. This figure highlights the key components and chemical modifications involved in the development of collagen matrices, fibrin-based hydrogels, PEG hydrogels, HA hydrogels, gelatin-chitosan hydrogels, GelMA hydrogels with rGO, and PLGA scaffolds. These synthesis strategies underscore the versatility of biomaterial design in creating angiogenesis-supportive environments.

#### Comparative analysis and application perspectives of composite biomaterials

In the vascularization of COs, GelMA hydrogels, PCL-PLGA composite scaffolds, and Gel-CS composite hydrogels each exhibit distinct advantages. GelMA hydrogels exhibit excellent biocompatibility and bioactivity. Their natural cell-adhesive motifs (e.g., RGD sequences) facilitate endothelial cell attachment, proliferation, and lumen formation. Furthermore, GelMA can be photo-crosslinked to enable precise 3D bioprinting of biomimetic vascular networks, making it suitable for constructing complex microvascular systems and co-culture models of cardiomyocytes and vascular cells. However, the use of animal-derived gelatin introduces concerns regarding batch-to-batch variability, immunogenicity, and potential viral contamination, limiting its clinical translatability. In addition, unmodified GelMA exhibits limited mechanical strength and cannot withstand the high mechanical stresses associated with beating COs.^147^^,^^148^

PCL-PLGA composite scaffolds integrate the high mechanical strength of PCL with the tunable degradability of PLGA. By adjusting their compositional ratios, these scaffolds can achieve precise control over mechanical properties and degradation kinetics, rendering them suitable for myocardial repair and large-scale cardiac patches. Nevertheless, the acidic byproducts generated during PLGA degradation may impair cell viability, and the material’s poor cell adhesion and limited intrinsic bioactivity remain challenges.

Gel-CS composite hydrogels combine favorable flexibility with significant bioactivity. They offer a supportive environment for cardiomyocytes and endothelial progenitor cells, and also possess inherent antibacterial properties, making them suitable for soft cardiac patches and vascularized microenvironments. However, their limited mechanical strength restricts their use in high-load myocardial regeneration scenarios. Additionally, the solubility and degradation rate of chitosan are highly pH-dependent, posing further challenges for consistent *in vivo* application.^149^

In summary, GelMA is well suited for the precise construction of microvascular networks and *in vitro* vascularization models; PCL-PLGA composites are more appropriate for structurally stable, degradable scaffolds in large-volume tissue repair; and Gel-CS hydrogels are ideal for soft patches supporting cell adhesion and angiogenesis. These materials should be selected and optimized based on specific application needs, considering their physical, chemical, and biological properties in an integrated manner.

To enable a systematic comparison of the advantages and limitations of various materials in the context of cardiac organoid vascularization, commonly employed natural, synthetic, and composite biomaterials are summarized in Table 1. Drawing upon a comprehensive review of the literature, this table delineates the key properties of each material. As evidenced by the comparison, factors such as biological performance, mechanical characteristics, degradation profiles, and biosafety play decisive roles in determining material suitability for specific tissue engineering applications and vascularization strategies. Therefore, in both experimental research and translational practice, the selection and optimization of biomaterial systems should be informed by the summarized attributes in accordance with the functional requirements and structural demands of the target tissue.

**Table 1:** Comparative summary of biomaterials in cardiac organoid vascularization

Material	Advantages	Limitations	Reference
Collagen	biocompatible, biodegradable; natural cell adhesion motifs (e.g., GFOGER sequences); promotes endothelial adhesion, migration, and vascular network formation	low mechanical strength; imprecisely regulated degradation rate; batch variability from natural sources; poor thermal and solvent stability; slowly biodegradable	^55^^,^^58^^,^^60^^,^^63^^,^^64^
Fibrin	biocompatible, biodegradable; ECM-like structure; good plasticity and *in situ* gelation; adjustable gelation kinetics; supports vascularization and provides cytoprotection	rapid degradation; weak mechanical strength; poor long-term structural integrity; batch variability due to blood-derived origin; potential risk of viral contamination	^65^^,^^66^^,^^69^^,^^70^^,^^71^^,^^74^^,^^85^^,^^86^
HA	high tissue affinity; immunomodulatory effects; easily modified chemically; suitable for delivering cells and growth factors; capable of activating multi-level signaling cascades to promote angiogenesis	high-molecular-weight HA may inhibit angiogenesis; poor physical stability; easily degraded; short tissue half-life; requires chemical crosslinking for stable structure	^76^^,^^77^^,^^79^^,^^81^^,^^82^^,^^83^^,^^84^
PEG	good biocompatibility and tunable mechanics; chemically modifiable; suitable for controlled delivery of cells and factors	lacks bioactivity; needs functionalization with biomolecules; limited biodegradability; risk of chronic inflammation upon long-term retention	^88^^,^^89^^,^^90^^,^^91^^,^^93^^,^^94^^,^^121^
PLGA	biocompatible, biodegradable; tunable degradation and release kinetics; good mechanical adjustability	degradation produces acidic byproducts, potentially cytotoxic; requires buffering strategies; poor cell adhesion	^95^^,^^96^^,^^97^^,^^98^^,^^100^^,^^101^^,^^102^^,^^103^^,^^104^^,^^105^^,^^106^^,^^107^^,^^108^^,^^109^^,^^110^^,^^111^
PCL	High mechanical strength; biodegradable; good biocompatibility; tunable scaffold architecture; suitable for delivering cells and bioactive factors	Slow degradation; not suitable for rapid remodeling; limited bioactivity, often requires combination with other materials or factors	^113^^,^^114^^,^^115^^,^^118^^,^^120^^,^^122^
GelMA	biocompatible and bioactive; contains RGD motifs promoting endothelial adhesion; supports 3D printing; can load angiogenic factors or exosomes	derived from animal-sourced gelatin with batch variability; immunogenic potential; poor mechanical strength in unmodified form; limited resistance to long-term stress	^123^^,^^125^^,^^126^^,^^127^^,^^128^^,^^129^^,^^130^^,^^147^^,^^148^
PCL-PLGA	high mechanical strength; amenable to surface modification; tunable composition for matching mechanical and degradation requirements	PLGA degradation products may affect cell viability; limited bioactivity; requires optimization of overall biological performance	^131^^,^^132^^,^^134^^,^^137^
Gelatin-Chitosan	injectable; good flexibility and bioactivity; supports cell adhesion and proliferation; exhibits antibacterial properties	low mechanical strength; poor load-bearing capacity; chitosan solubility and degradation sensitive to pH variation	^138^^,^^139^^,^^140^^,^^141^^,^^142^^,^^149^

## Strategies for promoting cardiac organoid vascularization with biomaterials

### Micro and nanotechnology

Micro-nano fabrication technology is an advanced manufacturing approach that enables the creation of complex structures and devices at micro- and nanoscale resolutions. This technology holds great promise in the biomedical field, particularly for constructing biomimetic vascular networks and promoting vascularization in organoids.^150^^,^^151^ As a critical subfield, 3D bioprinting allows for the integration of biomaterials and organoids with precise control over design, material composition, and chemical functionality to produce micro-nano architectures with specific biological functions.^151^ In vascular tissue engineering, micro-nano fabrication enables precise control over the size, shape, and spatial organization of vascular structures, effectively mimicking the microarchitecture of native vasculature. This capability offers essential tools for studying vascular physiology and pathology and provides novel strategies for tissue engineering and regenerative medicine.^152^ In recent years, 3D bioprinting has made substantial progress in constructing perfusable cardiac tissue models. For example, 3D printing can be used to fabricate scaffolds with specific microarchitectures that guide blood vessel development and promote vascularization in engineered tissues.^153^ Kupfer et al. developed a photo-crosslinkable bioink based on natural ECM components and used 3D bioprinting to fabricate a human cardiac tissue model containing two chambers and perfusion channels. Through *in situ* differentiation of hiPSCs within the construct, the model exhibited electromechanical integration, rhythmic contraction, and perfusion functionality.^154^ Similarly, Noor et al. printed patient-specific cardiac patches containing perfusable vasculature, which showed synchronized contractions and vessel fusion capabilities.^155^

Another promising micro-nano fabrication strategy for promoting vascularization in COs is electrospinning. This technique allows the fabrication of nanofibrous scaffolds with high surface area-to-volume ratios and tunable fiber diameters, closely mimicking the structural features of natural ECM. These scaffolds provide an ideal biophysical environment for endothelial cell adhesion, migration, and neovascularization.^156^ For instance, Tillman et al. fabricated tubular PCL/collagen electrospun nanofibrous scaffolds, which were seeded with ECs) and vascular SMCs for *in vivo* stability assessments. The results demonstrated that these cell-seeded scaffolds effectively supported EC and SMC adhesion and growth and exhibited favorable anti-thrombogenic properties.^157^ Additionally, Wang et al. incorporated optimized concentrations of calcium silicate into aligned chitosan-based nanofibers via electrospinning, demonstrating the synergistic potential of bioactive ions and nanostructured biomaterials in cardiovascular tissue engineering. The resulting composite cardiac patch scaffold enhanced the expression of angiogenic and cardiac markers, improved sarcomere organization, and promoted calcium signaling in neonatal rat cardiomyocytes.^158^

Microfluidics is another vital micro-nano fabrication approach that plays an increasingly important role in cardiac organoid vascularization. In recent years, microfluidic platforms have demonstrated unique advantages in constructing complex vascular networks and enabling 3D tissue perfusion *in vitro*. These devices can precisely control fluid dynamics to simulate the physical and chemical microenvironment of the native vasculature, supporting long-term organoid cultivation and vascular development. A recently developed universal microfluidic platform enabled the vascularization and perfusion culture of various 3D cell spheroids, including mesenchymal bodies, pancreatic islets, and stem-cell-derived vascular organoids. This system supported endothelial network formation and functional integration with endothelial-rich spheroids and vascular organoids, enabling continuous perfusion for up to 30 days and significantly enhancing organoid growth, maturation, and functionality.^159^ Furthermore, Wu et al. employed acoustofluidic techniques to spatially organize suspended endothelial cells within a microfluidic chip using standing acoustic waves. These cells subsequently underwent lumen formation and maturation within hydrogel matrices under interstitial flow, resulting in the successful construction of functional vascular networks with predefined geometry. The resulting “vessel-on-a-chip” model demonstrated robust perfusability and preserved endothelial barrier function, offering a high-resolution, reproducible, and biomimetic platform for vascular biology and regenerative medicine research.^160^

In summary, with the continuous advancement of micro-nano fabrication technologies, strategies such as 3D bioprinting, electrospinning, and microfluidics are being increasingly integrated to build physiologically relevant vascular networks within COs. These technologies not only improve the structural and functional sophistication of organoids but also provide critical support for disease modeling and clinical translation in regenerative medicine.

### Scaffold material design

Scaffold materials play a critical role in vascular tissue engineering, as the porosity, morphology, and arrangement of scaffolds significantly influence endothelial cell migration and the formation of microvascular networks. Scaffold porosity is one of the key factors affecting cell migration and tissue formation. High porosity provides more space for cell infiltration and proliferation while also facilitating nutrient transport and the removal of metabolic waste products. Scaffold materials for vascular tissue engineering include natural materials, biodegradable polymers, and composite materials, which typically possess a porous structure to support cell growth and tissue formation.^161^ Moreover, the study by Valentin et al. emphasized the importance of scaffold porosity in meeting cellular metabolic demands, noting that cells require sufficient gas and nutrient exchange with the surrounding microenvironment.^162^ The morphology and arrangement of scaffold materials can guide the direction and mode of cell migration. In Hamblin’s research, the authors suggested that the porous structure of scaffolds, with inherent nanoscale cavities, can accommodate cells and macromolecules, indicating that the microstructure of scaffolds can influence cellular behavior.^163^ Timnak et al.’s study further discussed the impact of micro- and nanostructured surfaces on cell adhesion and proliferation, highlighting that nanoscale surface structures can enhance cell adhesion and improve material biocompatibility.^164^ These features are crucial for guiding endothelial cell migration and microvascular network formation. The design and properties of scaffold materials are essential for promoting the formation of microvascular networks. Tissue survival in tissue engineering requires rapid vascularization, which is influenced by the choice of scaffold material and seeded cells.^165^ For example, PLGA and beta-tricalcium phosphate (β-TCP) have shown good angiogenic effects due to their material properties. Studies indicate that β-TCP scaffolds enhance the angiogenic response of MSCs compared to PLGA scaffolds, thereby accelerating vascularization and significantly improving blood vessel formation.^165^

In summary, the porosity, morphology, and arrangement of scaffold materials play a pivotal role in providing an appropriate microenvironment and physical guidance for endothelial cell migration and microvascular network formation, which is critical for the success of vascular tissue engineering. Carefully designed scaffold materials can promote cell migration, proliferation, and differentiation, thereby facilitating effective tissue repair and regeneration.

### Growth factor delivery

The use of biomaterials for the controlled release of angiogenic factors, such as VEGF, into organoids to promote vascularization is a key technique in tissue engineering and regenerative medicine. This approach enables the precise delivery of growth factors at specific locations and time points, thereby effectively promoting new blood vessel formation and tissue regeneration. First, the design and optimization of biomaterials can enable controlled release of VEGF and other growth factors. For example, by altering the inherent properties of biomaterials, the release behaviors of VEGF and other factors can be independently modulated, achieving a combination of rapid and sustained release.^166^ Furthermore, the use of different biomaterials, such as natural and synthetic polymer matrices, allows for local and controlled release of VEGF and other growth factors to promote angiogenesis.^167^ Second, biomaterials can facilitate the sequential delivery of multiple growth factors to enhance angiogenesis. For instance, the sequential release of VEGF and platelet-derived growth factor (PDGF) via biomaterials can promote the rapid formation of mature vascular networks.^168^ Additionally, co-delivery of VEGF and PDGF with low doses of bone morphogenetic protein-2 (BMP-2) can significantly enhance vascularized bone regeneration.^169^ The controlled release properties of biomaterials can also be achieved through various methods. For example, 3D printing technology can be used to co-deliver growth factors and cells within a biomaterial scaffold, simulating the natural wound healing process and promoting functional bone formation.^170^ Furthermore, nanoparticle-based carrier systems can enable spatiotemporal controlled release of growth factors, overcoming the challenges of rapid degradation and nonspecific distribution of growth factors *in vivo*.^171^ However, the controlled release of growth factors faces several challenges. For instance, growth factors typically have a short *in vivo* half-life, strong dose-dependence, and spatial and temporal activity limitations that hinder their clinical application.^172^ Additionally, excessive delivery of growth factors can result in severe side effects, such as vascular leakage and hypotension.^167^ Therefore, the development of intelligent biomaterials capable of precisely delivering growth factors, such as stimuli-responsive materials, is an important direction for future research.^173^ In conclusion, using the controlled release properties of biomaterials to deliver angiogenic factors like VEGF is an effective strategy to promote vascularization within organoids. By optimizing the design of biomaterials, precise delivery and controlled release of growth factors can be achieved, thereby enhancing new blood vessel formation and tissue regeneration. However, challenges associated with the controlled release of growth factors must be overcome to enable their widespread application in clinical treatments.

### Cell-assisted vascularization

Co-culturing cardiomyocytes (CMs), ECs, and supporting cells, along with using biomaterial scaffolds to guide cell organization, represents a promising strategy for promoting vascularization and improving the survival rate of transplanted tissues. This approach can mimic *in vivo* angiogenesis processes, facilitating the formation and maturation of new blood vessels, thereby enhancing blood supply and functional recovery in transplanted tissues. In several studies, co-culturing CMs and ECs (such as HUVECs) within biomaterial scaffolds has been shown to promote vascular network formation. For instance, in the study by Zieber, HUVECs, CMs, and cardiac fibroblasts (cardio-fibroblasts) were co-cultured within a scaffold, resulting in a vascular-like network structure. HUVECs were arranged in multi-layered fashion around the channels, while CMs were positioned between the channels and displayed characteristic features of mature cardiomyocytes.^174^ Supporting cells, such as MSCs, also play a crucial role in enhancing vascularization. In Shane Browne’s research, it was demonstrated that by designing semi-synthetic extracellular matrices (sECMs) with appropriate physical and biochemical signals, encapsulated supporting cells could be directed to differentiate and migrate in specific directions, thereby promoting vascular network formation.^175^ Additionally, supporting cells can further promote endothelial cell proliferation and migration through the secretion of growth factors and cytokines. Utilizing biomaterial scaffolds to guide the adhesion, proliferation, and vascular network formation of cardiomyocytes, endothelial cells, and supporting cells is critical. For example, in alginate scaffolds, microchannels can be created, and the presentation of adhesion peptides and angiogenic factors can encourage the formation of vascular-like networks within the construct.^174^ In the study by Matthias W Laschke, it was found that undifferentiated adipose-derived mesenchymal stem cell (adMSC) spheroids, as attractive units for vascularization, when seeded into polyurethane scaffolds, exhibited significant vascularization defects,^176^ indicating the necessity of appropriate biomaterials. Moreover, to promote the maturation and stability of the vascular network, endothelial cell sheets cultured under hypoxic conditions can enhance the vascularization of the cell sheets.^177^ Alternatively, introducing a sandwich structure in stem cell microtissues can promote spontaneous extension and connection of two separate layers of HUVECs to the stem cell layer, resulting in the formation of a functional vascular network that integrates with the host vascular system.^178^ In conclusion, co-culturing cardiomyocytes, endothelial cells, and supporting cells, along with using biomaterial scaffolds to guide the formation of stable vascular networks within organoids, is an effective strategy for enhancing tissue vascularization and improving the survival rate of transplanted tissues. This strategy simulates *in vivo* angiogenesis processes, promoting the formation and maturation of new blood vessels, thus improving blood supply and functional recovery of transplanted tissues. Future research may focus on exploring different cell combinations, biomaterial scaffolds, and culture conditions to optimize the formation and stability of vascular networks, ultimately improving the long-term survival and functional recovery of transplanted tissues.

**Figure 13:**
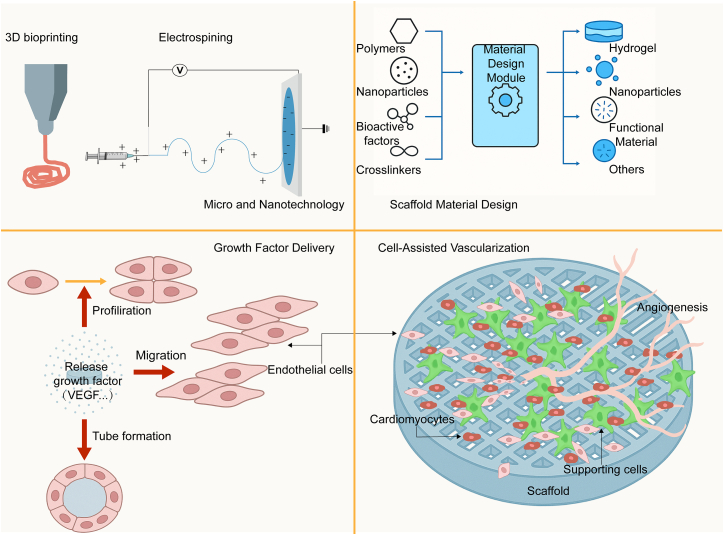
Schematic illustration of major strategies for promoting vascularization The diagram highlights several representative approaches, including micro/nanotechnology, scaffold design, growth factor delivery, and cell-assisted techniques, all aimed at enhancing vascular formation.

Figure 13 provides an integrated summary of the major approaches employed to promote vascularization in COs, including micro-nano technologies, biomaterial scaffold design, bioactive molecule delivery, and cellular strategies.

## Challenges and future prospects of biomaterials in cardiac organoid vascularization

### Material innovation

The vascularization of COs imposes stringent requirements on the degradation characteristics of scaffold materials, particularly with respect to synchronizing scaffold degradation with the dynamic process of neovascularization. Many current materials exhibit degradation profiles that are either too rapid or too slow. The former may lead to premature scaffold collapse and compromised organoid stability, whereas the latter can hinder vascular infiltration and maturation.^179^ Therefore, regulating the degradation behavior of biomaterials is essential for enhancing functional reconstruction of engineered cardiac tissues. On one hand, degradation tunability can be achieved by optimizing material composition and microstructure. For example, adjusting the ratio of natural to synthetic polymers or incorporating microporous architectures can improve permeability and cellular accessibility.^180^ Studies have shown that degradation products of natural materials such as collagen and HA exhibit favorable biocompatibility, reduce inflammatory responses, and facilitate tissue integration.^181^ On the other hand, functionalization of biomaterials has emerged as a key research focus. Incorporating bioactive factors (e.g., VEGF and bFGF) or peptide sequences (e.g., RGD) into scaffold materials can activate endothelial cell adhesion and migration, thereby promoting angiogenesis.^127^^,^^130^ Moreover, emerging “smart materials” that respond to external stimuli (e.g., pH, temperature, and light) or biological signals can achieve spatiotemporally controlled degradation and on-demand release of bioactive agents. Such materials offer significant potential in tissue engineering and provide precise microenvironmental modulation tools for organoid vascularization.^182^ Therefore, material innovation is essential for promoting cardiac organoid vascularization by addressing key challenges such as scaffold degradation, biocompatibility, and vascular integration. By designing tunable degradable materials, incorporating bioactive factors, and developing stimuli-responsive systems, researchers can better mimic the native cardiac microenvironment. These advances support synchronized scaffold degradation with vascular growth, enhance endothelial function, and reduce inflammation, ultimately improving organoid stability and functionality for regenerative applications.

### Biofabrication technologies

With the advancement of three-dimensional biomanufacturing technologies, the construction of structurally refined and functionally complex vascularized COs has become increasingly feasible. Among these, 3D bioprinting has emerged as a powerful tool in tissue engineering and organoid fabrication due to its high spatial resolution and ability for individualized assembly. By precisely controlling the spatial distribution of cells, biomaterials, and bioactive factors, this technique enables the biomimicry of native vascular architecture, thereby enhancing nutrient exchange and improving organoid viability.^183^ Beyond printing resolution, optimization of cellular sources and co-culture strategies—such as combinations of human amniotic epithelial cells (hAECs), HUVECs, and mesenchymal stem cells—has proven effective in promoting angiogenesis and stabilizing vascular structures.^184^ In addition, engineered microvasculature formed *ex vivo* can integrate with host vasculature *in vivo* via self-assembly or guided organization mechanisms.^185^ Bioreactor systems, which simulate physiological conditions like shear stress and oxygen gradients, further promote endothelial lineage commitment and vascular branching, improving perfusion capabilities.^184^ Collectively, the synergistic progress in 3D bioprinting, multicellular co-culture, and dynamic bioreactor platforms is expected to overcome current bottlenecks in cardiac organoid vascularization, supporting their functional maturation and scalable production.

### Clinical translation prospects

Biomaterials hold significant promise for clinical translation in the vascularization of COs; however, multiple challenges remain to be addressed. Although a variety of biomaterials have demonstrated impressive efficacy in promoting vascularization in laboratory settings, their successful transition to clinical application necessitates overcoming several hurdles. Chief among these are biocompatibility and long-term stability—particularly in the dynamic environment of the heart—where materials must sustain effective integration with host tissue and support prolonged vascular function.

Another major barrier lies in the scalability and standardization of biomaterial production. Current synthesis and processing technologies are often inadequate for large-scale manufacturing with consistent quality. Innovations in biofabrication, such as 3D bioprinting and automated production systems, are anticipated to play a pivotal role in addressing these limitations.^186^

From a regulatory perspective, biomaterials must undergo rigorous evaluations of safety and efficacy, conforming to national regulatory standards. To advance through clinical trials successfully, biomaterials must be manufactured under good manufacturing practice (GMP) conditions and validated in multi-phase studies across diverse patient cohorts. Moreover, the growing emphasis on precision medicine and personalized therapies could accelerate the development of patient-specific biomaterial solutions, broadening their application in cardiac organoid vascularization.^187^

Looking ahead, interdisciplinary collaboration spanning materials science, cell biology, engineering, and clinical medicine will be essential to overcome translational bottlenecks. With continued technological advancements, biomaterials designed for cardiac organoid vascularization are expected to mature into clinically viable platforms, offering new regenerative therapies for CVD.

## Acknowledgments

The authors would like to thank Sichuan Provincial People’s Hospital for providing the platform and resources that supported this work. For financial support, this work was supported by the Sichuan Provincial Science and Technology Program (2023YFS0036), Key Research and Development Project of Science and Technology of Sichuan Province (2022YFS0605), Sichuan Science and Technology Program (24NSFJ0271), Outstanding Youth Science Fund Project of Sichuan Natural Science Foundation (24NSFJQ0271) & Research Project of Affiliated Hospital of North Sichuan Medical College (2023LC006 and 2023ptzk022), and 10.13039/501100018542Natural Science Foundation of Sichuan Province (23NSFSC1540 and 23NSFSC1541).

## Author contributions

J.L., Y.F., Y.L., and P.Q. contributed equally to the conceptualization, writing, and review of the manuscript. J.S. and C.M. contributed to the literature review and drafting of sections. Q.L. and G.L. provided guidance on the structure and content of the review. L.Z. led the manuscript preparation and coordinated the review process. P.C. and B.Y. supervised the overall direction of the manuscript and reviewed the final version.

## Declaration of interests

The authors declare no competing interests.
